# The effect of liquid composition on the partitioning of Ni between olivine and silicate melt

**DOI:** 10.1007/s00410-016-1319-8

**Published:** 2016-12-19

**Authors:** Andrew K. Matzen, Michael B. Baker, John R. Beckett, Bernard J. Wood, Edward M. Stolper

**Affiliations:** 1 Division of Geological and Planetary Sciences, California Institute of Technology, Pasadena, CA 91125 USA; 2 Department of Earth Sciences, University of Oxford, Oxford, OX1 3AN UK; 3 Department of Earth Sciences, University of Oxford, Oxford, OX1 3AN UK

**Keywords:** Nickel partitioning, Olivine, Hawaii, Iceland, Ocean-island basalt

## Abstract

We report the results of experiments designed to separate the effects of temperature and pressure from liquid composition on the partitioning of Ni between olivine and liquid, $D_{\text{Ni}}^{\text{ol/liq}}$. Experiments were performed from 1300 to 1600 °C and 1 atm to 3.0 GPa, using mid-ocean ridge basalt (MORB) glass surrounded by powdered olivine in graphite–Pt double capsules at high pressure and powdered MORB in crucibles fabricated from single crystals of San Carlos olivine at one atmosphere. In these experiments, pressure and temperature were varied in such a way that we produced a series of liquids, each with an approximately constant composition (~12, ~15, and ~21 wt% MgO). Previously, we used a similar approach to show that $D_{\text{Ni}}^{\text{ol/liq}}$ for a liquid with ~18 wt% MgO is a strong function of temperature. Combining the new data presented here with our previous results allows us to separate the effects of temperature from composition. We fit our data based on a Ni–Mg exchange reaction, which yields $\ln \left( {D_{\text{Ni}}^{\text{molar}} } \right) = \frac{{ -\Delta _{r(1)} H_{{T_{\text{ref}} ,P_{\text{ref}} }}^{ \circ } }}{RT} + \frac{{\Delta _{r(1)} S_{{T_{\text{ref}} ,P_{\text{ref}} }}^{ \circ } }}{R} - \ln \left( {\frac{{X_{\text{MgO}}^{\text{liq}} }}{{X_{{{\text{MgSi}}_{ 0. 5} {\text{O}}_{ 2} }}^{\text{ol}} }}} \right).$ Each subset of constant composition experiments displays roughly the same temperature dependence of $D_{\text{Ni}}^{\text{ol/liq}}$ (i.e.,$-\Delta _{r(1)} H_{{T_{\text{ref}} ,P_{\text{ref}} }}^{ \circ } /R$) as previously reported for liquids with ~18 wt% MgO. Fitting new data presented here (15 experiments) in conjunction with our 13 previously published experiments (those with ~18 wt% MgO in the silicate liquid) to the above expression gives $-\Delta _{r(1)} H_{{T_{\text{ref}} ,P_{\text{ref}} }}^{ \circ } /R$ = 3641 ± 396 (K) and $\Delta _{r(1)} S_{{T_{\text{ref}} ,P_{\text{ref}} }}^{ \circ } /R$ = − 1.597 ± 0.229. Adding data from the literature yields $-\Delta _{r(1)} H_{{T_{\text{ref}} ,P_{\text{ref}} }}^{ \circ } /R$ = 4505 ± 196 (K) and $\Delta _{r(1)} S_{{T_{\text{ref}} ,P_{\text{ref}} }}^{ \circ } /R$ = − 2.075 ± 0.120, a set of coefficients that leads to a predictive equation for $D_{\text{Ni}}^{\text{ol/liq}}$ applicable to a wide range of melt compositions. We use the results of our work to model the melting of peridotite beneath lithosphere of varying thickness and show that: (1) a positive correlation between NiO in magnesian olivine phenocrysts and lithospheric thickness is expected given a temperature-dependent $D_{\text{Ni}}^{\text{ol/liq}} ,$ and (2) the magnitude of the slope for natural samples is consistent with our experimentally determined temperature dependence. Alternative processes to generate the positive correlation between NiO in magnesian olivines and lithospheric thickness, such as the melting of olivine-free pyroxenite, are possible, but they are not required to explain the observed correlation of NiO concentration in initially crystallizing olivine with lithospheric thickness.

**Electronic supplementary material:**

The online version of this article (doi:10.1007/s00410-016-1319-8) contains supplementary material, which is available to authorized users.

## Introduction

Unlike most other minor and trace elements, Ni is compatible in olivine (ol) over a broad range of temperatures, pressures, and silicate liquid (liq) compositions (i.e., $D_{\text{Ni}}^{\text{ol/liq}}$ = NiO^ol^/NiO^liq^ is greater than one, where NiO^*φ*^ refers to the concentration of NiO in phase *φ* by weight). One consequence of the compatibility of Ni in olivine and other mantle phases (e.g., Mysen 1978) is that the NiO contents of residual lherzolites are insensitive to low degrees of partial melting; this is consistent with the observed narrow distribution of the NiO contents of olivines from spinel lherzolites: 0.37 (median) ± 0.03 [mean absolute deviation (MAD)], 308 analyses (Korenaga and Kelemen 2000; Jun Korenaga, personal communication, 2011) and 0.37 ± 0.01, 172 analyses (Herzberg et al. 2013). Thus, unless the degree of melting is very high (e.g., >50%; Herzberg et al. 2016), one might reasonably expect that the first olivines to crystallize from a partial melt of a lherzolitic source would recover, within ~8%, the NiO content of olivine in the initially unmelted source (Herzberg et al. 2016). However, olivine phenocrysts from ocean islands often have NiO contents exceeding 0.5 wt% (e.g., Clague et al. 1991; Sobolev et al. 2005, 2007). Possible explanations for these elevated NiO concentrations in olivine—and the melts from which they are derived—include an olivine-free component in the mantle produced by metasomatism of peridotite by silicic partial melts of recycled oceanic crust (Sobolev et al. 2005, 2007), Ni-enrichment in the mantle source due to interaction with the Ni-rich core (Ryabchikov 2003; Herzberg et al. 2013), or a $D_{\text{Ni}}^{\text{ol/liq}}$ that is a function of temperature (*T*) and/or pressure (*P*), in addition to composition (Leeman and Lindstrom 1978; Mysen and Kushiro 1979; Li and Ripley 2010; Putirka et al. 2011; Matzen et al. 2013).

Our ability to discriminate among these hypotheses depends on knowing $D_{\text{Ni}}^{\text{ol/liq}}$ and, specifically, how $D_{\text{Ni}}^{\text{ol/liq}}$ is affected by variables such as temperature, pressure, oxygen fugacity (*f*O_2_), and olivine and liquid compositions. Experiments in most studies measuring $D_{\text{Ni}}^{\text{ol/liq}}$ for a given bulk composition have been performed at a single pressure; thus, any change in temperature results in a change in liquid composition as olivine is added or subtracted from the liquid. Interpreting the results of such experiments is not straightforward because changes in liquid composition, olivine composition, and temperature, or a combination of these factors could result in the observed changes in $D_{\text{Ni}}^{\text{ol/liq}}$ (Arndt 1977; Hart and Davis 1978; Takahashi 1978; Wang and Gaetani 2008; Putirka et al. 2011). Matzen et al. (2013) isolated the effects of pressure and temperature from those of composition by performing experiments in which pressure and temperature were changed in concert; this yielded coexisting olivine–liquid pairs with roughly constant olivine Mg#s (100 × Mg/[Mg + Fe], molar) of ~90, which we refer to as Fo_90_, and liquid compositions of ~18 wt% MgO. These experiments showed for a picritic liquid that temperature has a significant effect on $D_{\text{Ni}}^{\text{ol/liq}}$. Here, we present the results of similar series of experiments designed to determine whether the temperature dependence of $D_{\text{Ni}}^{\text{ol/liq}}$ is also a significant function of liquid composition, as predicted by Hirschmann and Ghiorso (1994).

## Experimental and analytical methods

**Fig. 1:**
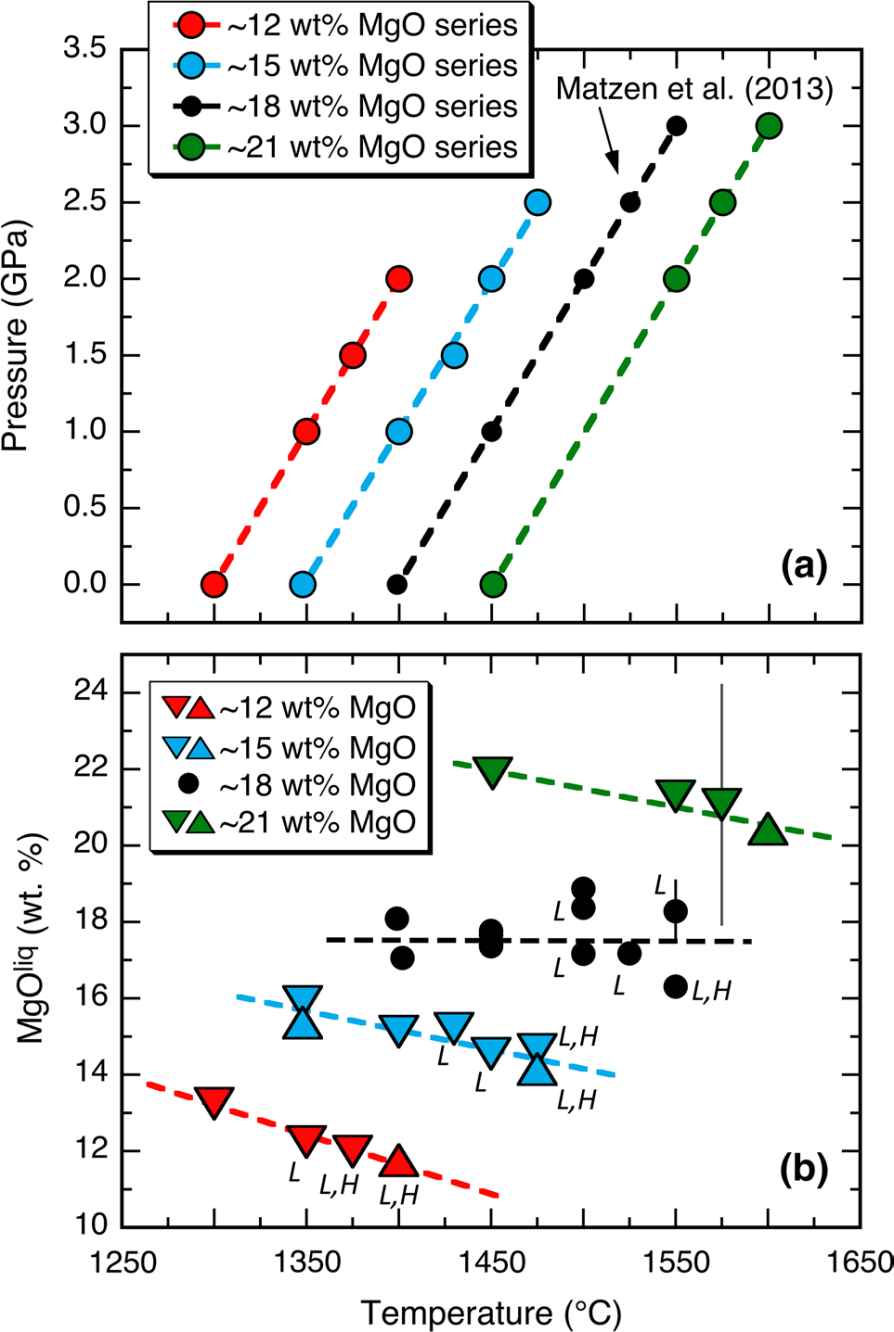
Experimental run conditions and melt compositions for experiments in this work and Matzen et al. (2013). **a** Pressure as a function of temperature. *Filled circles* and associated *dashed lines* correspond to series with similar glass compositions. **b** *Symbols* denote concentration of MgO (wt%) in glass as a function of temperature; *multiple symbols* at a given temperature for a specific MgO series reflect forward and reverse experiments at those *P*–*T* conditions (for this study, *downward* and *upward pointing triangles*, respectively); *dashed lines* associated with each MgO series are weighted least-squares fits. Low-Ca (L) and high-Ca (H) pyroxenes are indicated, where present in the run products. Error bars in this and succeeding figures are one sample standard deviation, unless otherwise stated, and are shown where larger than the size of the symbol. The large error bars on the 1575 °C experiment (~21 wt% MgO series) are a consequence of quench crystallization

Run conditions are given in Table 1 and shown graphically in Fig. 1a. We used the same experimental methods and materials as Matzen et al. (2013; see their Table 1 for a description of starting materials). Briefly, we performed high-pressure experiments by surrounding a small chip of fused Juan de Fuca MORB glass with powdered Kilbourne Hole olivine in a Pt–graphite double capsule. Experiments were run in two stages: (1) a filled capsule was sintered at a temperature below the estimated solidus of the basalt [based on the Pertermann and Hirschmann (2003) parameterization], effectively forming a low-permeability, polycrystalline olivine crucible; (2) the temperature was then increased to the final run temperature, followed by a pressure increase to the final run pressure. Except for 1 GPa experiments, sintering was conducted at pressures 0.1–0.35 GPa below the desired run pressure. For experiments at 1 GPa, we hot-pressed the capsule at 1.4 GPa, but in an effort to retain the relative sense of piston motion (i.e., hot piston-in), we slowly lowered the pressure from 1.4 GPa to below 1.0 GPa before the final temperature ramp, such that when the temperature was increased to its final value, the pressure would remain slightly below the desired value; finally, pressure was increased to the final value for the run. Note that in the first of two experiments where we employed this method (Run 51), the pressure was not lowered enough before the final temperature ramp; upon reaching the final run temperature, the pressure was too high and had to be lowered to its target value (hot piston-out). One-atmosphere experiments were conducted using methods similar to Wang and Gaetani (2008): mixtures of MORB glass and powdered Kilbourne Hole olivine were placed in a crucible fabricated from a single crystal of San Carlos olivine and suspended in a flowing mixture of CO_2_ and H_2_ that produced an *f*O_2_ ~1.7 log units below the QFM buffer, an *f*O_2_ similar to those measured in high-pressure graphite-capsule experiments (Médard et al. 2008); *f*O_2_s for all one-atmosphere experiments are listed in Table 1. The temperatures and pressures shown in Fig. 1a were selected so that the resulting liquids would have approximately 12, 15, and 21 wt% MgO (hereafter referred to as the ~12, ~15, and ~21 MgO^liq^ series). We were not entirely successful in meeting these target liquid compositions; all of our MgO^liq^ series show decreasing MgO^liq^ with increasing temperature (Fig. 1b). We conducted mostly forward experiments (downward pointing triangles in Fig. 1b) in which low-Ni glass (<0.03 wt% NiO) was equilibrated with Ni-bearing olivine (~0.37 wt% NiO). The four reversal experiments (upward pointing triangles in Fig. 1b; run numbers appended with an R in Table 1) used starting glasses doped with 1 wt% NiO (i.e., higher than in the olivine).

**Table 1:** Run conditions and run products

Run #	Series	Final run conditions			Hot press conditions			Capsule^a^	Initial glass^b^ (wt%)	Run products^c^	Phase proportions^d^ (wt%)	% NiO change^e^ (relative)	*Q*-value^f^	$D_{\text{Ni}}^{\text{ol/liq}}$ ^g^
		*T* (°C)	*P* (GPa)	*t* (h)	*T* (°C)	*P* (GPa)	*t* (h)							
49	15	1430	1.5	12.0	915	1.40	5.8	Pt-C	5.09	gl, ol, l-px	43.7, 52.9, 3.4	+1.3	0.87	5.30 (33)
50	15	1450	2.0	12.1	940	1.74	6.0	Pt-C	6.28	gl, ol, l-px	49.2, 36.0, 14.8	+2.1	1.97	5.34 (29)
51^h^	15	1400	1.0	12.0	915	1.40	6.0	Pt-C	4.51	gl, ol	38.0, 62.0	−0.6	0.07	5.91 (34)
52	12	1375	1.5	12.0	915	1.38	6.3	Pt-C	2.47	gl, ol, l-px, h-px	49.8, 35.1, 2.4, 12.7	+1.7	1.00	6.94 (46)
53	12	1350	1.0	12.2	915	1.40	6.7	Pt-C	3.19	gl, ol, l-px	45.1, 52.8, 2.1	+1.1	0.68	7.29 (52)
56	15	1475	2.5	12.0	965	2.20	5.9	Pt-C	1.78	gl, ol, l-px, h-px	41.1, 30.3, 7.5, 21.0	+1.5	0.99	4.94 (25)
58	21	1550	2.0	12.0	940	1.74	6.0	Pt-C	1.59	gl, ol	5.3, 94.7	−4.2	1.00	3.69 (16)
59	21	1575	2.5	12.0	965	2.20	6.0	Pt-C	1.08	gl^i^, ol	13.4, 86.6	+1.1	1.00	3.38 (59)
62R	12	1400	2.0	12.0	940	1.70	6.2	Pt-C	0.96	gl, ol, l-px, h-px	32.9, 35.5, 7.2, 24.3	–	1.00	7.01 (69)
63R	15	1475	2.5	12.2	965	2.23	6.0	Pt-C	2.13	gl, ol, l-px, h-px	41.7, 29.1, 4.2, 25.0	–	1.00	5.10 (17)
64R	21	1600	3.0	12.0	990	2.65	6.2	Pt-C	3.81	gl, ol	13.6, 86.4	–	0.88	3.35 (11)
65	15	1348	1 × 10^−4^	12.1	–	–	–	SCOl	−8.63	gl, ol	78.7, 21.3	−11.5	0.47	6.03 (38)
67R	15	1348	1 × 10^−4^	12.2	–	–	–	SCOl	−8.63	gl, ol	82.8, 17.2	–	0.55	5.89 (31)
68	12	1300	1 × 10^−4^	11.5	–	–	–	SCOl	−9.16	gl, ol	78.3, 21.7	−4.7	0.69	6.95 (44)
70	21	1451	1 × 10^−4^	8.1	–	–	–	SCOl	−7.66	gl, ol	51.4, 48.6	−6.2	0.57	3.89 (17)

Run number followed by the letter R denotes a reversal experiment. Relative change in bulk Na_2_O for one-atmosphere experiments, Runs 65, 67R, 68, and 70, are −15.5, −15.1, −13.8, and −16.2%, respectively. Hot press conditions are not applicable to the 1-atm experiments

^a^Capsule abbreviations: Pt-C, platinum–graphite double capsule; SCOl, San Carlos olivine

^b^Value listed for one-atmosphere experiments is the measured log_10_ *f*O_2_

^c^Run products abbreviations: gl = glass (quenched liquid) or a quench mat, ol = olivine, l-px = low-Ca pyroxene, h-px = higher-Ca pyroxene

^d^Phase proportions, calculated by mass balance, are given in the same order as listed in the run products column

^e^Relative change (in percent) of NiO from the bulk composition based on mass balance; negative sign denotes a decrease and a positive sign indicates an increase in the NiO content of the bulk. Values are not reported for the reversal experiments where NiO in the olivine is strongly zoned toward the rims leading to values that are a numerical artifact of this zoning (see text for further discussion)

^f^Goodness-of-fit measure, values above 0.05 indicate solutions acceptable at the 95% confidence level

^g^Measured olivine–liquid Ni partition coefficient, by weight. Number enclosed in parentheses represents the analytical uncertainty according to the least units cited, e.g., 5.30 (33) represents 5.30 ± 0.33. Error reflects the propagated analytical uncertainty (one sample standard deviation) of NiO in the olivine and glass (Table 2)

^h^Upon heating to the final run temperature, pressure increased above the desired final run–pressure. Pressure was slowly bled off until the final run–pressure was reached. Thus, this experiment was hot piston-out; all other high-pressure runs were hot piston-in

^i^Quench mat

Measurements of all phase compositions are reported in Table 2. Glass and olivine compositions were measured on Caltech’s JEOL JXA-8200 using analytical and data processing procedures described by Matzen et al. (2013). Pyroxenes were analyzed on Oxford’s JEOL 8600, using an accelerating voltage of 15 keV, a 40 nA beam current, and a 1 μm beam at locations ~10 μm from the edge of each pyroxene grain, utilizing a set of metal (Ni), oxide (Cr_2_O_3_), and mineral (wollastonite, rutile, jadeite, hematite, rhodonite, periclase, orthoclase) standards. On-peak counts were collected for 30–60 s with high and low backgrounds counted for half of the on-peak time. Pyroxene data acquired at Oxford were processed using the PAP matrix correction (Pouchou and Pichoir 1988). Pyroxenes from some of the runs were also analyzed at Caltech (processed using a modified ZAF correction: CITZAF, Armstrong 1988); for the Caltech data, most of the average pyroxene compositions overlap at one sigma with those obtained from the Oxford analyses and, in all cases, they overlap at two-sigma.

**Table 2:** Phase compositions

Run #	Phase	N	SiO_2_	TiO_2_	Al_2_O_3_	Cr_2_O_3_^a^	FeO	MnO	MgO	CaO	Na_2_O	K_2_O	P_2_O_5_	NiO^a^	Total
49	liq	10	48.71 (14)	1.68 (2)	12.23 (5)	0.028 (2)	10.14 (7)	0.20 (3)	15.21 (8)	9.27 (5)	2.34 (3)	0.15 (1)	0.16 (2)	0.070 (4)	100.18
49	ol	10	40.53 (8)	0.017 (5)	0.075 (3)	0.017 (3)	11.05 (8)	0.154 (4)	47.69 (7)	0.26 (1)	–	–	–	0.371 (6)	100.17
49	l-px	6	55.77 (18)	0.18 (3)	2.79 (9)	0.082 (12)	7.16 (28)	0.15 (2)	31.77 (26)	1.79 (8)	0.09 (1)	bdl	bdl	0.14 (2)	99.93
50	liq	10	46.44 (8)	1.86 (3)	12.58 (7)	0.022 (1)	11.05 (8)	0.19 (2)	14.55 (6)	10.04 (4)	2.54 (3)	0.17 (1)	0.18 (2)	0.072 (4)	99.68
50	ol	10	40.40 (15)	0.016 (4)	0.09 (2)	0.012 (4)	11.76 (13)	0.158 (6)	47.02 (26)	0.29 (2)	–	–	–	0.386 (5)	100.14
50	l-px	8	55.00 (41)	0.19 (2)	4.16 (51)	0.070 (18)	7.28 (14)	0.16 (2)	30.12 (37)	2.44 (7)	0.19 (1)	bdl	bdl	0.16 (3)	99.77
51	liq	10	49.98 (11)	1.59 (2)	12.01 (11)	0.028 (1)	9.93 (8)	0.175 (12)	15.13 (7)	8.69 (3)	2.21 (3)	0.141 (5)	0.15 (2)	0.063 (3)	100.10
51	ol	10	40.76 (6)	0.021 (6)	0.049 (5)	0.018 (3)	10.79 (5)	0.149 (7)	48.22 (17)	0.25 (1)	–	–	–	0.369 (7)	100.62
52	liq	10	48.11 (8)	1.93 (1)	14.12 (9)	0.022 (1)	10.49 (6)	0.167 (13)	11.99 (5)	10.35 (4)	2.86 (6)	0.19 (1)	0.19 (3)	0.054 (4)	100.46
52	ol	6	40.47 (7)	0.015 (9)	0.06 (2)	0.014 (4)	12.81 (24)	0.174 (6)	46.63 (17)	0.26 (4)	–	–	–	0.371 (3)	100.80
52	l-px	8	54.48 (35)	0.26 (4)	3.93 (61)	0.082 (19)	8.31 (14)	0.17 (3)	29.69 (32)	2.50 (12)	0.15 (3)	bdl	bdl	0.11 (2)	99.69
52	h-px	13	54.66 (62)	0.24 (6)	3.29 (81)	0.084 (22)	8.21 (35)	0.21 (3)	26.42 (1.04)	6.18 (96)	0.35 (5)	bdl	bdl	0.11 (3)	99.76
53	liq	15	50.06 (11)	1.78 (3)	13.56 (11)	0.025 (2)	9.29 (10)	0.17 (2)	12.24 (10)	10.12 (5)	2.57 (3)	0.16 (1)	0.16 (3)	0.049 (3)	100.18
53	ol	10	40.62 (9)	0.018 (6)	0.05 (1)	0.018 (2)	11.95 (10)	0.166 (5)	47.23 (20)	0.27 (3)	–	–	–	0.358 (9)	100.67
53	l-px	2	56.16 (60)	0.20 (5)	2.15 (58)	0.098 (9)	8.15 (5)	0.177 (5)	31.01 (5)	2.11 (3)	0.08 (2)	bdl	bdl	0.13 (2)	100.28
56	liq	10	44.81 (15)	2.23 (4)	12.96 (6)	0.016 (1)	12.06 (7)	0.19 (2)	14.65 (7)	9.24 (4)	2.73 (4)	0.20 (1)	0.22 (3)	0.078 (3)	99.39
56	ol	7	40.52 (18)	0.027 (3)	0.13 (2)	0.008 (3)	12.23 (60)	0.154 (6)	46.96 (61)	0.26 (2)	–	–	–	0.387 (11)	100.69
56	l-px	10	53.43 (39)	0.23 (2)	6.01 (51)	0.047 (9)	7.74 (18)	0.16 (1)	29.31 (31)	2.38 (32)	0.28 (4)	bdl	bdl	0.13 (3)	99.74
56	h-px	7	52.58 (42)	0.32 (6)	6.41 (55)	0.053 (11)	7.38 (32)	0.19 (3)	23.41 (70)	7.97 (64)	0.85 (5)	bdl	bdl	0.11 (2)	99.30
58	liq	7	48.12 (13)	1.23 (2)	9.12 (8)	0.027 (1)	11.50 (13)	0.22 (3)	21.29 (10)	6.33 (5)	1.59 (5)	0.121 (5)	0.10 (2)	0.096 (2)	99.74
58	ol	10	41.03 (8)	0.011 (5)	0.084 (3)	0.015 (2)	9.61 (3)	0.131 (6)	49.28 (9)	0.17 (1)	–	–	–	0.355 (14)	100.70
59	liq^b^	10	48.03 (88)	1.31 (15)	9.55 (98)	0.025 (1)	11.56 (29)	0.22 (2)	21.07 (3.13)	6.85 (86)	1.60 (15)	0.12 (1)	0.11 (3)	0.111 (19)	100.57
59	ol	10	41.24 (8)	0.012 (6)	0.10 (1)	0.011 (2)	9.57 (3)	0.132 (5)	49.50 (7)	0.19 (1)	–	–	–	0.375 (10)	101.13
62R	liq	9	45.15 (10)	2.54 (3)	14.83 (6)	0.015 (4)	11.05 (9)	0.16 (3)	11.76 (7)	9.15 (4)	3.46 (11)	0.27 (1)	0.28 (2)	0.232 (11)	98.90
62R	ol	8	39.90 (11)	0.031 (5)	0.12 (3)	0.010 (3)	13.32 (21)	0.168 (5)	44.75 (25)	0.27 (1)	–	–	–	1.625 (138)	100.18
62R	l-px	8	52.38 (50)	0.41 (9)	6.83 (60)	0.052 (21)	8.66 (42)	0.16 (1)	27.60 (74)	2.50 (56)	0.28 (7)	bdl	bdl	0.64 (8)	99.56
62R	h-px	12	51.12 (34)	0.52 (10)	7.54 (38)	0.062 (20)	7.93 (45)	0.20 (3)	19.72 (96)	10.39 (76)	0.92 (6)	bdl	bdl	0.66 (25)	99.10
63R	liq	9	44.38 (10)	2.33 (4)	13.15 (6)	0.010 (1)	12.01 (7)	0.19 (2)	14.15 (7)	9.20 (5)	2.82 (5)	0.22 (1)	0.27 (4)	0.279 (6)	99.00
63R	ol	7	40.36 (8)	0.033 (8)	0.13 (3)	0.006 (4)	12.75 (6)	0.157 (6)	45.70 (11)	0.28 (1)	–	–	–	1.425 (35)	100.85
63R	l-px	9	53.25 (72)	0.26 (4)	6.34 (70)	0.030 (12)	7.87 (17)	0.16 (2)	28.75 (40)	2.24 (14)	0.28 (2)	bdl	bdl	0.56 (4)	99.76
63R	h-px	8	52.58 (61)	0.32 (6)	6.69 (92)	0.054 (24)	7.91 (41)	0.20 (3)	21.87 (89)	8.23 (77)	0.94 (5)	bdl	bdl	0.73 (7)	99.56
64R	liq	10	46.74 (13)	1.40 (2)	9.64 (7)	0.024 (2)	11.60 (5)	0.21 (3)	20.45 (8)	7.44 (4)	1.70 (3)	0.127 (4)	0.15 (2)	0.197 (3)	99.67
64R	ol	10	41.00 (11)	0.016 (6)	0.14 (1)	0.012 (2)	9.69 (2)	0.133 (4)	48.94 (15)	0.225 (4)	–	–	–	0.660 (18)	100.82
65	liq	10	48.92 (14)	1.56 (3)	11.48 (8)	0.027 (1)	11.31 (9)	0.20 (3)	15.91 (6)	8.99 (5)	1.76 (4)	0.10 (1)	0.13 (3)	0.062 (4)	100.45
65	ol	10	40.98 (9)	0.017 (8)	0.019 (5)	0.024 (4)	10.77 (4)	0.153 (7)	48.52 (12)	0.22 (3)	–	–	–	0.373 (9)	101.09
67R	liq	10	48.91 (10)	1.55 (3)	11.41 (4)	0.028 (2)	11.55 (7)	0.20 (2)	15.37 (7)	9.05 (5)	1.81 (2)	0.11 (1)	0.14 (2)	0.370 (5)	100.49
67R	ol	9	40.57 (13)	0.017 (4)	0.021 (8)	0.027 (3)	10.88 (6)	0.155 (5)	46.78 (22)	0.24 (2)	–	–	–	2.178 (113)	100.86
68	liq	10	49.72 (17)	1.71 (4)	12.42 (6)	0.027 (2)	11.09 (7)	0.20 (2)	13.24 (6)	9.71 (7)	1.95 (3)	0.12 (1)	0.14 (3)	0.052 (3)	100.37
68	ol	10	40.68 (19)	0.020 (5)	0.017 (6)	0.021 (2)	12.03 (35)	0.171 (7)	47.33 (45)	0.22 (5)	–	–	–	0.361 (7)	100.84
70	liq	10	47.28 (14)	1.27 (2)	9.24 (7)	0.029 (1)	11.64 (4)	0.20 (2)	21.89 (12)	7.28 (5)	1.42 (3)	0.07 (1)	0.11 (3)	0.102 (4)	100.52
70	ol	4	41.22 (5)	0.008 (6)	0.040 (2)	0.019 (1)	8.74 (10)	0.123 (8)	49.93 (9)	0.22 (1)	–	–	–	0.396 (6)	100.70

All compositions listed in wt%. Phase abbreviations as in Table 1. Numbers in parentheses are analytical uncertainties in terms of the least units cited, e.g., 48.71 (14) corresponds to 48.71 ± 0.14 where 48.71 represents the average of* N* analyses, and 0.14 is one sample standard deviation of those analyses; when the error is ≥1.0, we include the decimal point. FeO* = all Fe as FeO. Dashed line indicates that the element was not analyzed, bdl = below detection limit

^a^Element in glass phase analyzed with a 200 nA beam current. Number of analyses used to determine concentrations in the glass for these elements are 12 for Run 75; 9 for Runs 50 and 53; 7 for Runs 61, and 62R; 5 for Runs 55 and 58; and 10 for all others

^b^Quench mat

In a reversal experiment, the NiO content of the starting glass is higher than that of a silicate melt in equilibrium with the surrounding olivine. The mass of olivine is large compared to the mass of liquid and NiO diffuses from the liquid into the surrounding olivine over the course of the experiment. Olivines that crystallize from the Ni-rich melt at the start of an experiment will have high NiO contents, but the Ni content at their rims decreases with time as the melt continues to lose Ni to the surrounding olivine. Our experiments were never run long enough to fully homogenize all phases, so olivines in contact with a melt pool are strongly zoned near their rims. In an effort to capture the equilibrium partition coefficient, we performed a series of analyses with 1–2 μm step sizes approaching the olivine–glass interface and interpreted the equilibrium NiO^ol^ to be the last point that showed no contamination from the glass (e.g., Matzen et al. 2013). No such procedure was conducted for the pyroxenes; reported NiO concentrations in pyroxenes from reversal experiments are most likely overestimates of the equilibrium values and they should not be used in the calculation of equilibrium partition coefficients.

## Experimental results

### Phase compositions

#### Glass

Matzen et al. (2013) produced a set of experiments with ~18 wt% MgO^liq^. In this work, we produced three additional series of experiments, each with a roughly constant liquid composition but offset from the next series by ~3–4 wt% MgO. Experiments were conducted at 1 atm and 1–3 GPa and spaced at 25–50 °C intervals (see Table 1; Fig. 1a). All of our experiments produced glasses with MgO contents within 1.2 wt% of their specified target value, but there are within-series variations. For example, in the ~12 wt% MgO^liq^ series, the 1-atm experiment produced a glass with 13.2 wt% MgO because the selected run temperature was too high, whereas other glasses from this series (11.8–12.2 wt% MgO) are much closer to the target of 12% (Fig. 1b). Similarly, Run 70 in the ~21 wt% MgO^liq^ series produced a glass with 21.9% MgO, higher than the others in the series (20.4–21.3 wt%). Below, we discuss the extent to which these deviations impact our evaluation of whether or not the temperature dependence of $D_{\text{Ni}}^{\text{ol/liq}}$ is a significant function of composition. As shown in Fig. 1b, MgO^liq^ decreases in all three experimental series (this study) with increasing temperature. In addition, the ~12 and ~15 MgO^liq^ series (and the ~18 wt% MgO^liq^ series; Matzen et al. 2013) all exhibit increases in incompatible element concentrations and decreases in SiO_2_ contents with increasing pressure (Table 2). Some variation in melt chemistry within an MgO^liq^ series reflects deviations in the run conditions from those needed to produce the desired melt composition in a run saturated with olivine. As discussed in Matzen et al. (2013), however, we infer that changes in silica largely reflect the crystallization of progressively larger amounts of pyroxene, which has higher SiO_2_ contents than the pyroxene-free glasses, (51–56 vs. 47–50 wt%), as the pressure of the experiments increases (see Table 2; Fig. 1b). Pyroxene crystallization is also likely at the root of increasing concentrations of incompatible elements in the melt with increasing pressure and temperature (see Matzen et al. 2013). As MgO in the liquid is lowered, pyroxene saturation occurs at progressively lower temperatures and pressures. For example, the 2.0 GPa run (1400 °C) in the ~12 wt% MgO^liq^ series crystallized 31.5 wt% low- and high-Ca pyroxene, whereas the 3.0 GPa run (1600 °C) in the ~21 wt% MgO series had only olivine and melt.

In general, it was difficult to quench high-MgO liquids to a homogeneous glass in our piston-cylinder apparatus, despite using the pressure–drop quench technique of Putirka et al. (1996). Run 59 (Table 2; ~21 wt% MgO^liq^ series; 2.5 GPa, 1575 °C) underwent substantial quench crystallization. We analyzed the quench mats using a 20-μm beam but were unable to reconstruct the melt composition without large errors (Fig. 1b). Even larger errors for quench mats in experiments at ~25 wt% MgO^liq^ led us to reject them. In addition, experiments at 1.0 and 1.5 GPa experiments in the ~21 wt% MgO^liq^ series yielded small melt pools that hindered the formation of a homogeneous glass, so no results are reported for these conditions.

#### Olivine

One feature of our experimental design is that the mass of an experimental charge is dominated by the starting olivine (Table 1); as a result, Mg#s of the olivine in our experiments are expected to vary little from that of the starting olivine (~90; see Table 1 in Matzen et al. 2013). If, however, the melt interacts with a relatively smaller volume of olivine directly adjacent to the melt (e.g., in the 1-atm experiments), the Mg#s of the near-melt olivine are more strongly influenced by the melt composition; for example, at 1 atm, the ~12 and ~15 wt% MgO^liq^ series melts are in equilibrium with olivines with slightly lower Mg#s (87.5 and 88.7, respectively). In all series, the Al_2_O_3_ contents of near-melt olivines increase with increasing temperature and pressure; for example, in the ~21 wt% MgO^liq^ series, Al_2_O_3_ contents increase from 0.04 at one atmosphere and 1450 °C to 0.14 at 3.0 GPa and 1600 °C. Olivine–liquid Al partition coefficients measured in our experiments agree well with the predictions of Agee and Walker (1990).

#### Pyroxene

Low-calcium pyroxene (1.8–2.5 wt% CaO) is observed in the ~12 and ~15 wt% MgO^liq^ series experiments, appearing at and above 1.0 and 1.5 GPa, respectively (see Table 1; Fig. 1b). Subcalcic pyroxenes (6.2–10.4 wt% CaO) are also present in the ~12 wt% MgO^liq^ series above 1.0 GPa and in the ~15 wt% MgO^liq^ series above 2.0 GPa. Na_2_O contents of low-Ca pyroxenes increase from 0.1 to 0.3 wt% with increasing temperature and pressure. The Al_2_O_3_ contents of both low- and high-Ca pyroxenes also increase with increasing temperature and pressure, from 2 to 8 wt% in the ~12 wt% MgO^liq^ series and from 3 to 7 wt% in the ~15 wt% MgO^liq^ series. These changes in the Na_2_O and Al_2_O_3_ contents in the pyroxenes are consistent with previous experimental measurements of the pressure and temperature dependence of Na_2_O and Al_2_O_3_ partitioning between pyroxene and silicate melt (e.g., Blundy et al. 1995; Frei et al. 2009). As mentioned briefly above, the SiO_2_ contents of the pyroxenes (51–56 wt%) are higher than those of liquids from pyroxene-free experiments in each series (47–50 wt% SiO_2_). Thus, the crystallization of pyroxene acts to reduce the SiO_2_ content of the glass.

### Mass balance

As described in Matzen et al. (2013), phase proportions in our experiments were determined using the nonlinear approach of Albarède and Provost (1977) and account for the variable amount of interaction between melt and the enclosing olivine; results are given in Table 1. All mass balance solutions are acceptable at the 95% confidence level (e.g., Press et al. 1992), suggesting that variations in liquid composition are well explained by the observed phases and their compositions (Table 2).

Table 1 includes the calculated change in bulk NiO for the forward experiments. For our piston-cylinder experiments, NiO was conserved in the central melt pool—changes range from −4.2 to +2.1% relative (negative number indicates a decrease). Losses of NiO in 1-atm experiments were larger (−4.7 to −11.5%). We did not mass balance NiO in the reversal experiments as olivines in contact with melt are strongly zoned with respect to Ni.

### Attainment of equilibrium

**Fig. 2:**
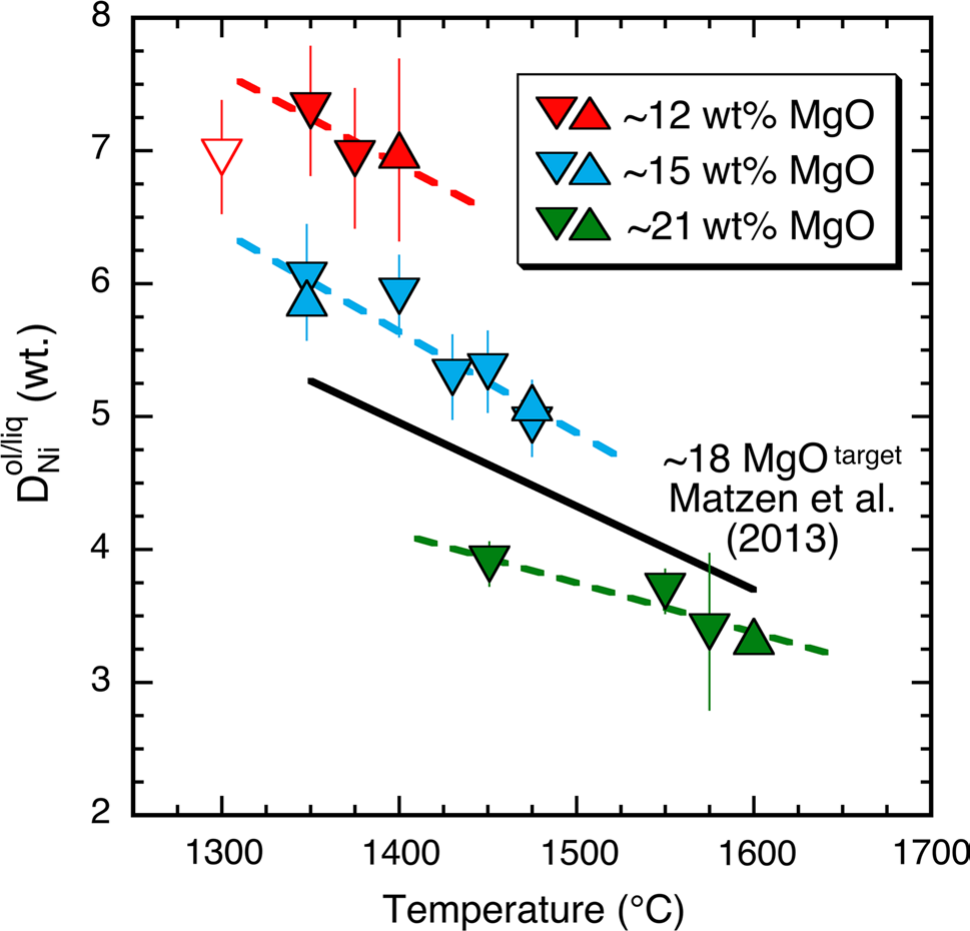
$D_{\text{Ni}}^{\text{ol/liq}}$ (by weight) versus temperature for the new experiments presented here and those of Matzen et al. (2013), which are represented by the *solid black line* (a weighted least-squares fit). *Downward* and *upward pointing triangles* represent forward and reversal experiments, respectively; *error bars* are propagated from the analytical uncertainties reported in Table 2. The *open symbol* is for a run (#68) deemed to have too magnesian a liquid composition to be included with the ~12 wt% MgO series experiments (see Supplemental Information for further discussion). *Dashed lines* are weighted least-squares fits to each respective series from this study. As discussed in the text, the slopes of all four lines are the same within uncertainty

To demonstrate a close approach to equilibrium, we conducted forward and reversal experiments. As in Matzen et al. (2013), a forward experiment is conducted with a low-NiO glass (<0.03 wt% NiO) embedded in olivine (0.37 wt% NiO). Thus, at the beginning of the experiment, the apparent $D_{\text{Ni}}^{\text{ol/liq}}$ is high, and over the course of the experiment, Ni moves from the olivine into the liquid (measured glasses from these experiments have 0.05–0.15 wt% NiO) and, thus, the apparent $D_{\text{Ni}}^{\text{ol/liq}}$ *decreases* toward the equilibrium value (and, thus, these forward experiments are indicated by downward pointing triangles in Figs. 1, 2). The reversal experiments were conducted using a glass doped with ~1 wt% NiO, similarly surrounded by powdered olivine with 0.37 wt% NiO. In these experiments, the initial apparent $D_{\text{Ni}}^{\text{ol/liq}}$ is low, and over the course of the experiment, NiO moves from the melt into the olivine so that the apparent $D_{\text{Ni}}^{\text{ol/liq}}$ *increases* (reversals are shown as upward pointing triangles in Figs. 1, 2). Where forward and reversal experiments were conducted at the same nominal temperature and pressure, the measured $D_{\text{Ni}}^{\text{ol/liq}}$ values overlap at one sigma, suggesting that the olivine–liquid Ni partition coefficients reported here closely approach equilibrium values (Fig. 2).

As stated in the Introduction, a goal of this work is to evaluate whether or not the temperature dependence of $D_{\text{Ni}}^{\text{ol/liq}}$ along the join MORB-Fo_90_ olivine is a significant function of composition. Such a dependency would be manifested in a different slope for $D_{\text{Ni}}^{\text{ol/liq}}$ versus temperature for each of the constant composition series. Figure 2 shows $D_{\text{Ni}}^{\text{ol/liq}}$ values of the ~12, ~15, and ~21 wt% MgO series experiments from this study (Tables 1, 2) along with weighted regression lines for each of these series and the ~18 wt% MgO series of Matzen et al. (2013). The reason for not including the lowest temperature run (open triangle) in the ~12 wt% series regression is discussed in detail in Supplemental Information.

Qualitatively, Fig. 2 reinforces the well-known observation that as MgO^liq^ increases, $D_{\text{Ni}}^{\text{ol/liq}}$ decreases (e.g., Hart and Davis 1978; Kinzler et al. 1990). However, the salient feature of Fig. 2 for present purposes is that the slopes of all four lines (~12, ~15, ~18, and ~21 wt% MgO^liq^) are statistically identical at 2 standard errors (SE): −0.70 ± 1.63/100 °C, −0.76 ± 0.21/100 °C, −0.63 ± 0.10/100 °C, and –0.37 ± 0.14/100 °C, respectively (listed uncertainties are 1 SE). The much larger uncertainty associated with the ~12 wt% MgO^liq^ series reflects the relatively larger errors in $D_{\text{Ni}}^{\text{ol/liq}}$ and the limited range in temperature. The statistical overlap among the four slopes suggests that, for bulk compositions along the join MORB-Fo_90_ olivine, the temperature dependence of $D_{\text{Ni}}^{\text{ol/liq}}$ is consistent with being independent of composition, although the progressive decrease in slope in going from liquids with ~15 to ~21 wt% MgO could reflect a small compositional dependence that would be broadly consistent with the calculations of Hirschmann and Ghiorso (1994) on the activity coefficient of NiSi_0.5_O_2_ in silicate liquids. Keeping in mind that increasing temperature can be associated with increasing pressure, the results shown in Fig. 2 also confirm the conclusion of Matzen et al. (2013) that, for constant liquid and olivine compositions, $D_{\text{Ni}}^{\text{ol/liq}}$ decreases with increasing temperature and pressure. In the following section, we fit our partitioning data using a thermodynamically inspired model that accounts for temperature and compositional effects on $D_{\text{Ni}}^{\text{ol/liq}}$ and we show that all but one of the 28 experiments that comprise the four MgO series are consistent with a single regression line in 1/*T* (K) space.

## Discussion

### Modeling $D_{\text{Ni}}^{\text{ol/liq}}$: ideal exchange reaction

Following Matzen et al. (2013) and many others (e.g., Hart and Davis 1978; Leeman and Lindstrom 1978; Kinzler et al. 1990), we fit our data using a Ni–Mg exchange reaction:

1 $${\text{MgSi}}_{0.5} {\text{O}}_{2}^{\text{ol}} + {\text{NiO}}^{\text{liq}} \leftrightarrow {\text{NiSi}}_{0.5} {\text{O}}_{2}^{\text{ol}} + {\text{MgO}}^{\text{liq}} ,$$

for which we write the following expression:

2 $$\Delta _{r(1)} G_{{{\it{T}},{\it{P}}}}^{ \circ } = - RT\ln \left( {\frac{{X_{{{\text{NiSi}}_{ 0. 5} {\text{O}}_{ 2} }}^{\text{ol}} X_{\text{MgO}}^{\text{liq}} }}{{X_{{{\text{MgSi}}_{ 0. 5} {\text{O}}_{ 2} }}^{\text{ol}} X_{\text{NiO}}^{\text{liq}} }} \times \frac{{\gamma_{{{\text{NiSi}}_{ 0. 5} {\text{O}}_{ 2} }}^{\text{ol}} \gamma_{\text{MgO}}^{\text{liq}} }}{{\gamma_{{{\text{MgSi}}_{ 0. 5} {\text{O}}_{ 2} }}^{\text{ol}} \gamma_{\text{NiO}}^{\text{liq}} }}} \right),$$

where $\Delta _{r(1)} G_{{{\it{T}},{\it{P}}}}^{{^\circ }}$, the standard-state Gibbs free energy change of reaction (1) at temperature (*T*, in Kelvin) and pressure (*P*, in bars), is independent of composition. $X_{i}^{\varphi }$ and $\gamma_{i}^{\varphi }$ are the mole fraction and activity coefficient of the *i*th component in phase *φ*, using the molar components of Matzen et al. (2013), and *R* is the gas constant. One of the benefits of an exchange reaction is that, over moderate ranges in temperature and pressure, $\Delta _{r(1)} C_{\it{p}}^{ \circ }$ and $\Delta _{r(1)} V$ are small enough to be safely neglected in the computation of $\Delta _{r(1)} G_{{{\it{T}},{\it{P}}}}^{{^\circ }}$, so that the standard-state changes in enthalpy and entropy for reaction (1) can be approximated as constants and the effect of pressure on $D_{\text{Ni}}^{\text{ol/liq}}$ is small [see Matzen et al. (2013) for a more detailed exposition of this important point]. Furthermore, at high temperatures, olivine can be treated as an ideal solution (e.g., Nafziger and Muan 1967; Campbell and Roeder 1968) and, as discussed by O’Neill and Eggins (2002) and O’Neill and Berry (2006) among others, the ratios of activity coefficients for divalent cations in silicate liquids can be approximated as a constant. Using these assumptions and defining a molar partition coefficient, $D_{\text{Ni}}^{\text{molar}} = \frac{{X_{{{\text{NiSi}}_{ 0. 5} {\text{O}}_{ 2} }}^{\text{ol}} }}{{X_{\text{NiO}}^{\text{liq}} }}$, Eq. (2) simplifies to

3 $$- \frac{{\Delta _{r(1)} H_{{T_{\text{ref}} ,P_{\text{ref}} }}^{ \circ } }}{RT} + \frac{{\Delta _{r(1)} S_{{T_{\text{ref}} ,P_{\text{ref}} }}^{ \circ } }}{R} = \ln \left( {D_{\text{Ni}}^{\text{molar}} } \right) + \ln \left( {\frac{{X_{\text{MgO}}^{\text{liq}} }}{{X_{{{\text{MgSi}}_{ 0. 5} {\text{O}}_{ 2} }}^{\text{ol}} }}} \right),$$

which is presented as Eq. (5) in Matzen et al. (2013). They fit their data and data from the literature to Eq. (3) and showed that the Ni–Mg exchange reaction has a significant temperature dependence. Although not discussed here, exchange reactions between Mg and other divalent cations (e.g., Mn and Fe^2+^) show little temperature dependence (Roeder and Emslie 1970; Matzen et al. 2011, 2013), making Ni unusual in this respect. Since previous work suggested a strong effect of liquid composition on activity coefficients of the NiSi_0.5_O_2_ component in silicate melts (Hirschmann and Ghiorso 1994), we seek to determine the extent to which compositional contributions to the temperature dependence of the Ni–Mg exchange reaction can be ignored (i.e., that the ratio of MgO^liq^ and NiO^liq^ activity coefficients is approximately constant).

**Fig. 3:**
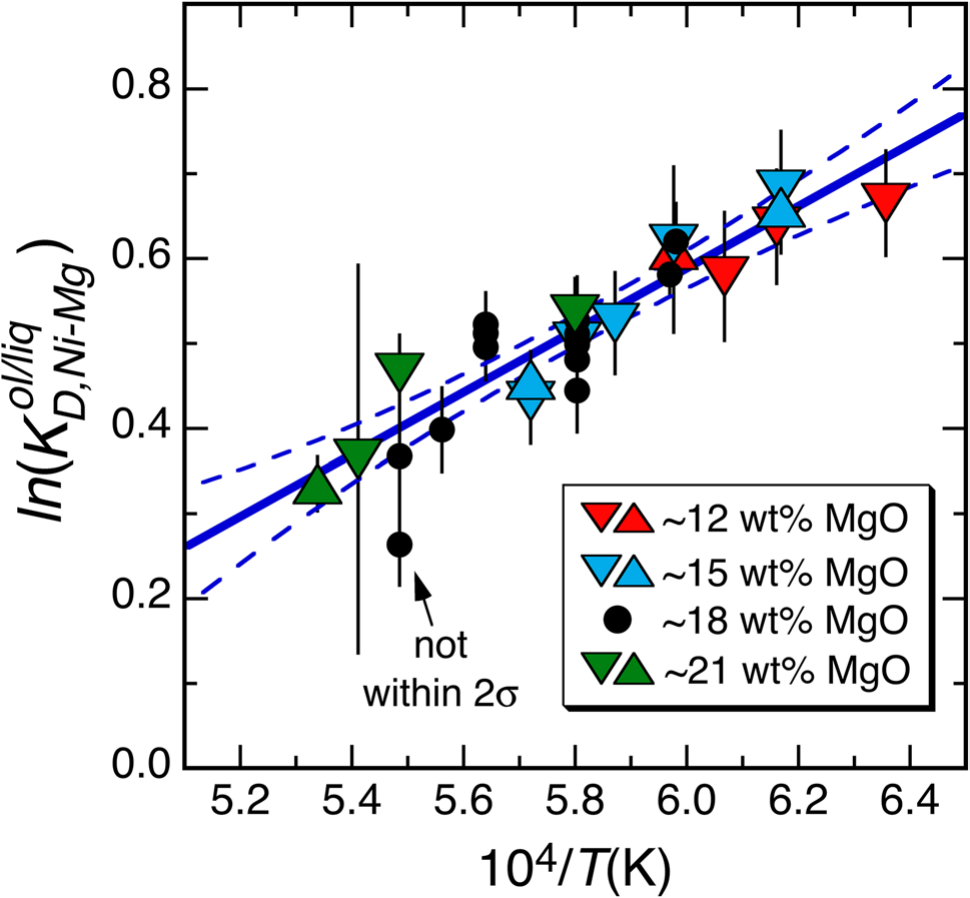
$\ln \left( {K_{{D,{\text{Ni}} - {\text{Mg}}}}^{\text{ol/liq}} } \right)$ versus 10^4^/*T*(K) showing experiments from this work (*downward* and *upward pointing triangles* denoting forward and reverse experiments, respectively) and Matzen et al. (2013) (*filled black circles*); *error bars* are propagated from the analytical uncertainties reported in Table 2 (this work) and Table 3 of Matzen et al. (2013). *Solid blue line* is a weighted least-squares fit to all of the data; *dashed blue curves* are the 95% confidence interval on the regression. Values of the slope and intercept for the *blue line* are 3641 ± 396 and −1.597 ± 0.229, respectively

**Fig. 4:**
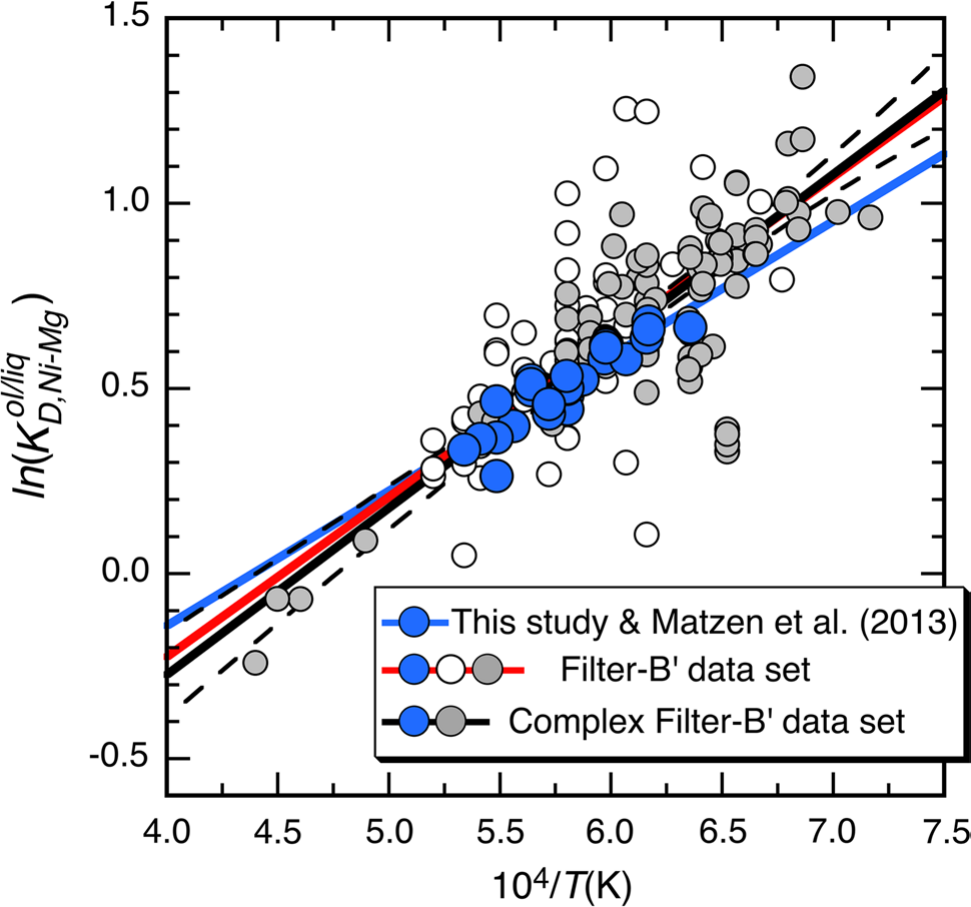
Values of $\ln \left( {K_{{D,{\text{Ni}} - {\text{Mg}}}}^{\text{ol/liq}} } \right)$ from this study and from the literature (the Filter-B data set from Matzen et al. 2013) plotted as a function of 10^4^/*T*(K); note that the Filter-B data set includes the experiments reported in Matzen et al. (2013). *Solid blue*, *red*, and *black lines* are fits to three different sets of data: this study + Matzen et al. (*filled blue circles*; *solid blue line* is the same as the fit shown in Fig. 3); Filter-B′ = this study + Matzen et al. (*filled blue circles*) + all other experiments in the Filter-B data set (*open* and *filled gray circles*; the fit is shown as a *red line*); Complex Filter-B′ = this study + Matzen et al. (*filled blue circles*) + a subset of the Filter-B data, culled so as to only include experiments with liquids containing at least SiO_2_, MgO, and ≥2 wt% each of CaO and Al_2_O_3_ (*filled gray circles*; the *open circles* are those literature experiments with <2 wt% CaO and/or Al_2_O_3_ in the glass). Fits to the Filter-B′ and Complex Filter-B′ data sets are based on a robust (iterative, bisquare-weighted, least-squares) technique and yield $-\Delta _{r(1)} H_{{T_{\text{ref}} ,P_{\text{ref}} }}^{ \circ } /R$ = 4321 ± 190 (K) and $\Delta _{r(1)} S_{{T_{\text{ref}} ,P_{\text{ref}} }}^{ \circ } /R$ =− 1.953 ± 0.115 (*solid red line*); $-\Delta _{r(1)} H_{{T_{\text{ref}} ,P_{\text{ref}} }}^{ \circ } /R$ = 4505 ± 196 (K) and $\Delta _{r(1)} S_{{T_{\text{ref}} ,P_{\text{ref}} }}^{ \circ } /R$ =− 2.075 ± 0.120 (*solid black line*), respectively. The *dashed black curves* are the 95% confidence bounds on the Complex Filter-B′ fit (for clarity, confidence bounds on the other two fits are not shown)

Figure 3 shows $\ln \left( {K_{{D,{\text{Ni}} - {\text{Mg}}}}^{\text{ol/liq}} } \right) = \ln \left( {D_{\text{Ni}}^{\text{molar}} } \right) + \ln \left( {\frac{{X_{\text{MgO}}^{\text{liq}} }}{{X_{{{\text{MgSi}}_{ 0. 5} {\text{O}}_{ 2} }}^{\text{ol}} }}} \right)$ plotted against inverse temperature for experiments from this work and Matzen et al. (2013). Although the exchange reaction is calculated using molar units (Figs. 3 and 4), using oxide weight percents yields identical values because the conversion factors for moles to weights cancel, a point that we return to in the context of Eqs. (4) and (5) below. The solid blue line in Fig. 3 is a weighted least-squares fit to all of the data (dashed curves represent the 95% confidence bounds). The data clearly define a nonzero slope, which can be equated to $-\Delta _{r(1)} H_{{T_{\text{ref}} ,P_{\text{ref}} }}^{ \circ } /R$ via Eq. (3) so that the Ni–Mg olivine exchange reaction is temperature dependent. Significantly, all of the data, with one exception (see Fig. 3), overlap the best-fit line at 2*σ* (75% of the 28 points overlap at 1*σ*), indicating that the experimental data (with MgO^liq^ contents that vary by nearly a factor of two) are consistent with single values of $-\Delta _{r(1)} H_{{T_{\text{ref}} ,P_{\text{ref}} }}^{ \circ } /R$ and $\Delta _{r(1)} S_{{T_{\text{ref}} ,P_{\text{ref}} }}^{ \circ } /R$. Further, all constant composition series exhibit statistically equivalent temperature dependencies (i.e., slopes for the ~12, ~15, and ~21 wt% MgO^liq^ series (this study) and the ~18 wt% MgO series (Matzen et al. 2013), all overlap at 1 SE: 2161 ± 2598; 5000 ± 1075; 3943 ± 712, and 4248 ± 1166 (K), respectively). In our discussion of Fig. 2, we separated out the lowest temperature experiment in the ~12 MgO^liq^ series, but this is included in Fig. 3 because differences in MgO content of the melt are accounted for by the term that includes $X_{\text{MgO}}^{\text{liq}}$ in Eq. (3). Note also that the slope reported here for the ~18 wt% MgO^liq^ experiments of Matzen et al. (2013), 3943 ± 712, differs from that reported by Matzen et al. (2013), 4375 ± 1050. Here, fits are weighted by errors on $K_{{D,{\text{Ni}} - {\text{Mg}}}}^{\text{ol/liq}}$, whereas Matzen et al. (2013) used an iterative, bisquare-weighted least-squares fit. When the errors on all data are known, using weights that are proportional to the inverse square of the compositional errors should result in superior estimates of the fit parameters (including uncertainties), compared to a bisquare-weighted fit. As noted above, the blue line in Fig. 3 is a weighted fit to all of our results simultaneously (i.e., to a combination of the new results presented here plus our previously published experiments with ~18 wt% MgO in the melt); $-\Delta _{r(1)} H_{{T_{\text{ref}} ,P_{\text{ref}} }}^{ \circ } /R$ and $\Delta_{r(1)} S_{{T_{\text{ref}} ,P_{\text{ref}} }}^{ \circ } /R$ for this best-fit line are 3641 ± 396 (K) and −1.597 ± 0.229, respectively, and *both* temperature ($- \Delta_{r(1)} H_{{T_{\text{ref}} ,P_{\text{ref}} }}^{ \circ } /R$ is nonzero) and the compositions of olivine and liquid affect $D_{\text{Ni}}^{\text{ol/liq}}$. If the assumptions made in deriving Eq. (3) are correct, we expect that the standard-state parameters obtained from fitting individual constant composition series will be identical to each other within uncertainty and, as noted above, this expectation is met.

In Fig. 4, our results are plotted together with a compilation of literature data. The Filter-B data set (from Matzen et al. 2013), which uses analytical totals (98.5–101.5 wt%) and Ni loss (<65%) as filtering criteria, has been updated to include data from this study; we refer to this amended data set as Filter-B′. Fitting the exchange reaction to the Filter-B′ data set (using an iterative, bisquare-weighted least-squares technique since not all studies report compositional uncertainties) gives a temperature dependency (4321 ± 190 K; 183 experiments; red line in Fig. 4) that overlaps within 2 SE of the slope obtained for the high-precision data shown in Fig. 3 (3641 ± 396 K; 28 experiments; the blue line in Fig. 4).

The Filter-B′ data set displays considerable scatter in Fig. 4. Some of this may be due to analytical errors or disequilibrium, but it is important to emphasize that this data set spans a large compositional range. For example, glass SiO_2_, Al_2_O_3_, FeO* (all Fe as FeO), CaO, and Na_2_O contents range from 40.5 to 68.8, 0 to 21.5, 0 to 37.2, 0 to 29.1, and 0 to 11.1 wt%, respectively, and olivine Mg#s range from 36 to 100. In comparison, glasses from this study and Matzen et al. (2013) are more restricted: SiO_2_, Al_2_O_3_, FeO*, CaO, and Na_2_O contents range from 44.4 to 50.2, 9.1 to 14.8, 9.3 to 12.4, 6.3 to 10.4, and 1.4 to 3.5 wt%, respectively, and olivine Mg#s ranging from 86 to 91. Given the broad range in bulk and phase compositions, it is possible, indeed likely, that the simplifications we made in arriving at Eq. (3)—especially that the ratio of activity coefficients for MgO^liq^ and NiO^liq^ is constant—do not hold for all of the literature experiments. Ghiorso and Sack (1995) commented on this issue when they stated that “…workers who have focused their effects on thermodynamic modeling of simple melt systems (see Berman and Brown 1987, for a synthesis and review) have demonstrated that quite complicated models are required to achieve the same level of data reproducibility as simpler formulations… provide in magmatic [natural] liquids.” Unfortunately, quantifying the extent to which the ratio of MgO^liq^ and NiO^liq^ activity coefficients varies for all the experimental liquids in the Filter-B′ data set is difficult. Although there has been substantial work on determining how NiO^liq^ activity coefficients vary with temperature and liquid composition, most of the experiments have been conducted using the 1-atm diopside–anorthite eutectic as a base composition, and thus, it is unclear whether a global fit to those data (e.g., Wood and Wade 2013) is applicable to the much wider range of liquid compositions encompassed by the Filter-B′ compilation. A similar situation exists for estimating MgO^liq^ activity coefficients; the activity model of Snyder and Carmichael (1992) is calibrated on natural silicate melts, and the activity models of Berman (1983) are tied to compositions in the system CaO–MgO–Al_2_O_3_–SiO_2_ (CMAS). Motivated by Ghiorso and Sack (1995), we note that many of the outliers in Fig. 4 are experiments from systems where Al_2_O_3_ or CaO are either absent or present at very low concentrations, <2 wt%, in the liquid (open symbols in Fig. 4). Therefore, we also show a refit to the Filter-B′ data set (black line; Fig. 4) after removing those 53 experiments where the bulk composition did not contain at least CMAS + NiO and the liquid ≥2 wt% each of Al_2_O_3_ and CaO (labeled Complex Filter-B′ on Fig. 4). Note that the scatter is reduced (the mean percent error on $D_{\text{Ni}}^{\text{ol/liq}}$ for the Complex Filter-B′ data set is 9.6% compared to 11.3% for Filter-B′), but the slope of the line (4505 ± 196; 130 experiments) overlaps at 1 SE with that based on the entire Filter-B′ data set (4321 ± 190) and at 2 SE for the fit obtained from the experiments in this study combined with those of Matzen et al. (2013), 3641 ± 396. Since our goal is to generate a simple predictive tool to model $D_{\text{Ni}}^{\text{ol/liq}}$ in natural olivine-bearing liquids as a function of temperature and melt composition, we prefer the Complex Filter-B′ fit in Fig. 4. In the Supplemental Information, we provide a more detailed discussion of the quality of the fit of Eq. (3) to the experimental data as well as a comparison with the temperature-independent $D_{\text{Ni}}^{\text{ol/liq}}$ model of Beattie et al. (1991). Finally, although Matzen et al. (2013) concluded that ∆*V* for the exchange reaction (Eq. 1) was small enough to ignore, we briefly revisited this issue by explicitly including a ∆*V* term in our fit to the Complex Filter-B′ data set ($\Delta C_{p}^{ \circ }$ was assumed to be zero). Consistent with the conclusions of Matzen et al. (2013), ∆*V* is small, −0.024 ± 0.015 J/mol, and the improvement in the fit is not statistically significant at the 95% confidence level.

Rearranging Eq. (3), transforming molar units to oxide weight percents (a simple replacement for exchange reactions), and substituting in our preferred values of $-\Delta _{r(1)} H_{{T_{\text{ref}} ,P_{\text{ref}} }}^{ \circ } /R$ and $\Delta_{r(1)} S_{{T_{\text{ref}} ,P_{\text{ref}} }}^{ \circ } /R$, yields an expression:

4 $$D_{\text{Ni}}^{\text{ol/liq}} = \exp \left[ {\frac{4505 \pm 196}{T} - 2.075 \pm 0.120 - \ln \left( {\frac{{\text{MgO}^{\text{liq}} }}{{\text{MgO}^{\text{ol}} }}} \right)} \right]$$

that can be used to calculate $D_{\text{Ni}}^{\text{ol/liq}} ,$ provided we have estimates of temperature and the MgO contents of the olivine and melt. Generally, the composition of the melt and the Mg# of olivine are known or assumed, and therefore, the corresponding temperature is readily determined using expressions explicit in temperature (e.g., Beattie 1993; Putirka et al. 2007) or any of a number of petrological programs, including MELTS (Ghiorso and Sack 1995) and Petrolog3 (Danyushevsky and Plechov 2011).

### The influence of lithospheric thickness on the NiO contents of olivine phenocrysts

In this section, we explore the influence that a temperature-dependent $D_{\text{Ni}}^{\text{ol/liq}}$ has on olivines erupting onto lithosphere of varying thickness. McKenzie and Bickle (1988), Ellam (1992), and Fram and Lesher (1993) provided early discussions of the potential influence of lithospheric thickness on the composition of erupted magmas. More recently, Sobolev et al. (2007) noted that lavas from areas with thick lithosphere (>70 km, e.g., Hawaii) tend to have magnesian olivines with higher NiO concentrations than those erupted onto thin lithosphere (e.g., Iceland) and to have higher concentrations than the values in mantle olivine (e.g., ~0.37 wt%; Korenaga and Kelemen 2000; Herzberg et al. 2013). They rationalized these observations as being a consequence of adiabatically rising parcels of mantle consisting of thermally (but not chemically) equilibrated packages of eclogite/pyroxenite and peridotite. As the mantle adiabatically ascends, the more fertile eclogite domains are the first to extensively melt (e.g., Hirschmann and Stolper 1996; Stolper and Asimow 2007; Lambart et al. 2016) and the resulting silica-rich liquids react with the surrounding peridotite to produce zones of olivine-poor to olivine-free, Ni-rich metasomatic pyroxenites. With continued upwelling, both the pyroxenite and the peridotite partially melt (although with different productivities) resulting in primary lavas that are mixtures of liquids originating from these two lithologies. The lithosphere limits the final pressure of partial melting so that melts produced beneath a thick lithosphere are dominated by those from the metasomatic pyroxenite; partial melts of these pyroxenites are Ni rich both because the pyroxenites are rich in Ni relative to most known mantle-derived pyroxenites (e.g., France et al. 2015) and also because these olivine-poor source rocks have low bulk *D*_Ni_ values, and thus, the olivines that precipitate from these melts are consequently predicted to be anomalously Ni rich. In contrast, partial melts produced beneath a thin lithosphere are diluted by more extensive contributions of melt from the peridotite, thus diluting the influence of the Ni-rich melts produced by melting of pyroxenite and resulting in olivine phenocrysts from shallow melting that are Ni poor relative to those from deeper melting. In the Sobolev et al. (2007) model, the composition of the mantle source (i.e., the relative amounts of pyroxenite), the mantle potential temperature, and the thickness of the lithosphere all influence the NiO contents of olivine phenocrysts crystallized from mantle-derived basalts. It is important to stress that this model, in the context of Ni contents of magnesian olivine phenocrysts, is driven by the assumption that $D_{\text{Ni}}^{\text{ol/liq}}$ is a function only of composition—given such an assumption, it is difficult to generate olivine phenocrysts with Ni contents significantly higher than those of olivine in the mantle source by low degrees of high-pressure partial melting followed by low-pressure crystallization.

Recently, a number of authors (Li and Ripley 2010; Niu et al. 2011; Putirka et al. 2011; Matzen et al. 2013) have suggested an alternative explanation for high-NiO olivine phenocrysts in localities such as Hawaii. These authors proposed that the crystallization of such olivine from basalts at low-pressure results from a temperature (and/or pressure) dependent $D_{\text{Ni}}^{\text{ol/liq}}$. In this class of models, a thick lithosphere imposes a large difference in temperature and pressure between a partial melt equilibrating with the mantle (generally presumed to be peridotitic) and the near-surface environment in which olivines begin to crystallize. Thus, it is the effect that lithospheric thickness has on the temperature and pressure difference between the conditions of melt segregation in the mantle and the cooler near-surface conditions at which forsteritic olivine phenocrysts grow that controls their NiO contents and that larger temperature differences lead to higher NiO contents in early crystallized olivines.

As noted above, Matzen et al. (2013) showed that the effect of pressure on $D_{\text{Ni}}^{\text{ol/liq}}$ is negligible for depths down to ~100 km (i.e., from 0 to 3 GPa)—a depth range that encompasses much of the variations in thickness of the oceanic lithosphere (e.g., Artemieva 2011). With our new olivine–melt partitioning data, we can test the hypothesis that a temperature-dependent $D_{\text{Ni}}^{\text{ol/liq}}$ and partial melting of a peridotitic source control the NiO contents of near-surface olivine phenocrysts. To compute an expected difference between the NiO content of a primary olivine phenocryst and lithospheric thickness, we use

5 $$\frac{{\text{NiO}^{\text{ol}} @T_{{1{-}{\text{bar}}}} }}{{\text{NiO}^{\text{ol}} @T_{\text{m}} }} = \exp \left[ {\frac{{-}{\Delta _{r(1)} H_{{T_{\text{ref}} ,P_{\text{ref}} }}^{ \circ } }}{R}\left( {\frac{1}{{T_{{1\text{-}{\text{bar}}}} }} - \frac{1}{{T_{\text{m}} }}} \right)} \right],$$

after Eq. (8) from Matzen et al. (2013), except that here, NiO^ol^ is in weight units. This equation is derived from two statements of Eq. (3) for $D_{\text{Ni}}^{\text{ol/liq}}$ involving two different temperatures: one for a primary olivine-saturated melt in the mantle source at a temperature of *T*_m_, and a second, for the same melt, at its 1 bar olivine-saturated liquidus, *T*_1-bar_, both converted to a weight basis. Given the temperature at which olivine begins to crystallize at low pressure (*T*_1-bar_) and assuming: (1) the Ni content of mantle olivine in the source region; (2) that the mantle melt last equilibrated at the base of the lithosphere; and (3) that the melt composition remained constant between the source and the site of phenocryst growth (and thus that the initial low-pressure olivine has the same Mg# as that in the mantle source, i.e., the olivine–liquid Fe^2+^–Mg exchange reaction is not a strong function of *P* and *T*; Toplis 2005), we can obtain *T*_m_ using the slope of the olivine-saturated liquidus (55 °C/GPa, Sugawara 2000). Equation (5) can then be used to predict the NiO content of initially crystallizing near-surface olivines as a function of lithospheric thickness. As discussed in Matzen et al. (2013), the slope of the olivine-saturated liquidus estimated by Sugawara (2000) is very similar to values derived from MELTS (Ghiorso and Sack 1995) and calculations from Eq. (4) of Putirka et al. (2007); temperatures at 3 GPa obtained by applying the polynomial “pressure-correction” of Herzberg and O’Hara (2002) to the 1-atm olivine–liquid geothermometer of Beattie (1993) are within 15 °C of those calculated using Sugawara’s 55 °C/GPa.

We assume that mantle olivines in the source region have 0.37 ± 0.03 wt% NiO (median ± MAD, mean absolute deviation; data for spinel lherzolites from Korenaga and Kelemen 2000; Jun Korenaga, personal communication, 2011), a value that overlaps with that for olivines from abyssal peridotites, 0.38 ± 0.03 (median ± MAD) (Hamlyn and Bonatti 1980; Shibata and Thompson 1986; Bonatti et al. 1992; Edwards and Malpas 1996; Ghose et al. 1996; Stephens 1997; Hellebrand et al. 2002; Brunelli et al. 2003; Seyler et al. 2003; Rampone et al. 2004; Warren and Shimizu 2010). Note that the spinel lherzolites considered by Herzberg et al. (2013) have an identical median but a smaller uncertainty (MAD = 0.01); in the following discussion, we use the error calculated using the Korenaga and Kelemen (2000) data set, as it is based on nearly twice as many analyses and reflects near global coverage. For a lithospheric thickness of zero, there is no temperature contrast between source and surface, so Eq. (5) recaptures the NiO content of the mantle olivine (i.e., 0.37 wt% NiO). With a thicker lithosphere, there is a temperature difference between the source and olivine saturation near the surface and our calculations predict that these low-pressure olivines will have higher NiO than those present in the mantle source region. Although it is the difference between *T*_m_ and *T*_1-bar_ in Eq. (5) that leads to differences in the Ni contents of the low-pressure olivines that crystallize from primary melts, it is inverse temperatures that appear in Eq. (5) and this means that variations in the 1-bar liquidus temperature (reflecting variations in primary melt composition, principally MgO content) have, as we show below, only a small effect on calculated Ni in low-pressure olivines. We used estimates of mantle potential temperature, *T*_P_, at mid-ocean ridge and hot-spot settings (~1325–1570 °C; Iwamori et al. 1995; Kinzler 1997; Ito et al. 1999; Asimow and Langmuir 2003; Courtier et al. 2007; Herzberg and Asimow 2008; Lee et al. 2009; Brown and Lesher 2014; Gale et al. 2014; Shorttle et al. 2014) (but see Putirka et al. 2007 for much higher estimates) and the expression from Herzberg and Asimow (2015) relating *T*_P_ to a 1-bar liquidus temperature to acquire an estimate for the range in *T*_1-bar_ values, 1266–1447 °C (with a midpoint of 1356 °C), which most likely covers the vast majority of low-pressure liquidus temperatures for primary basaltic and picritic magmas (e.g., Herzberg et al. 2007).

**Fig. 5:**
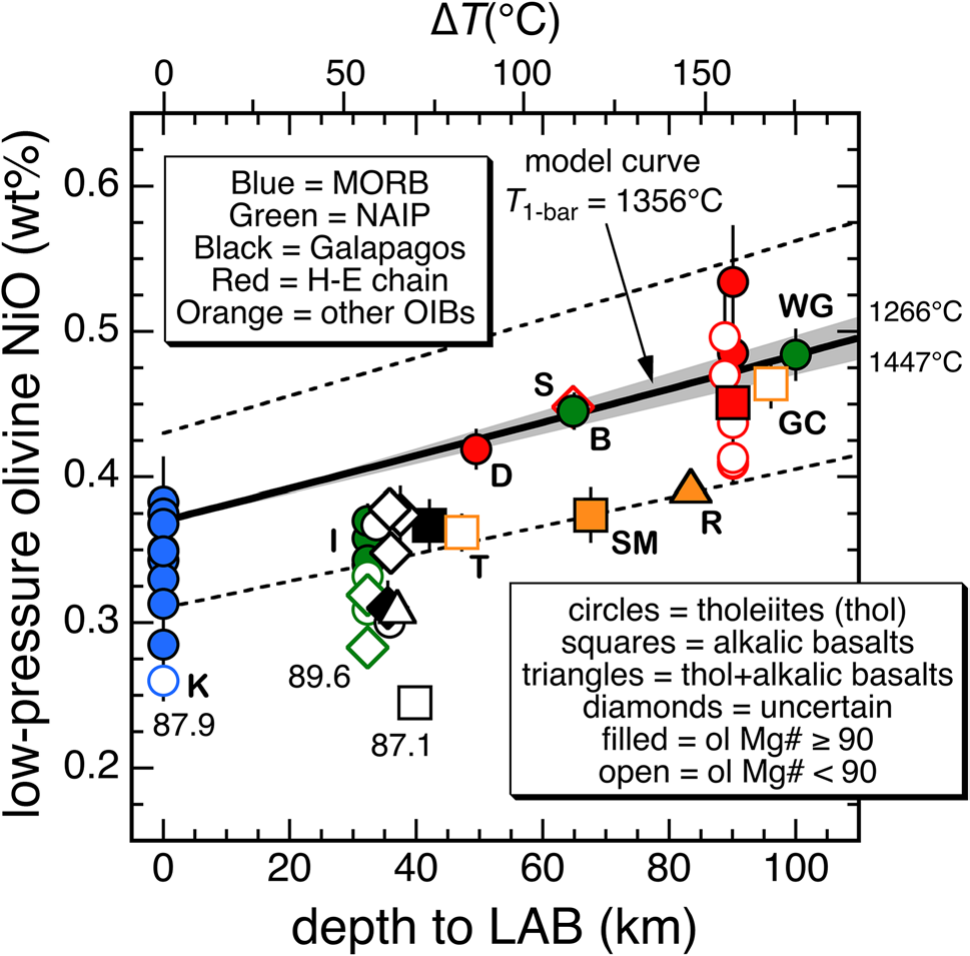
Maximum NiO contents of olivine phenocrysts versus depth to the lithosphere–asthenosphere boundary (km) at the time of eruption (see Table S1). The maximum NiO^ol^ content for each suite was calculated by substituting the Mg# for the most Mg-rich olivine at a given locality into the equation for a linear or power law fit to the Mg#^ol^ versus NiO^ol^ data for that suite; coefficients are reported in Table S2. Sites are differentiated by type of volcanism (tholeiitic, alkalic, or uncertain when the assignment was ambiguous; see notes to Table S1) and by Mg# (greater or less than 90). Values adjacent to the lowest MORB (mid-ocean ridge basalt), Iceland, and Galápagos points are the maximum olivine Mg#s at those three localities. *Abbreviations B* Baffin Island/Bay, *D* Detroit seamount (Emperor chain), *GC* Gran Canaria (Canary Islands), *H-E* Hawaiian–Emperor Chain, *I* Iceland, *K* Knipovich Ridge, *NAIP* North Atlantic Igneous Province, *OIB* ocean island basalt, *R* Réunion, *S* Suiko seamount (Emperor chain), *SM* Sao Migel (Azores), *T* Terceria (Azores), *WG* West Greenland. *Solid line* represents calculated enrichments in 1-bar primary olivine phenocrysts using Eq. (5) and assuming: a 1-bar liquidus temperature of 1356 °C, a linear relationship between liquidus temperature and pressure (and depth), and a mantle olivine NiO content of 0.37 wt%; *gray triangular band* denotes the effect of using the low and high estimates of 1-bar liquidus temperatures (1266 and 1447 °C) calculated from the range of mantle potential temperature estimates (see text); *dashed lines* represent how variations in mantle olivine NiO (±0.06 wt%) affect the 1356 °C curve. *Tick marks* along the top of the figure denote ∆*T* = *T*_m_ − *T*_1-bar_, where *T*_m_ is the temperature (and depth) at which the primary melt separates from the mantle; see text for further discussion

In Fig. 5, the solid black line was obtained using the above midpoint 1-bar liquidus temperature, 1356 °C in Eq. (5) coupled with the linear relationship in pressure between *T*_1-bar_ and the temperature of melt segregation from the mantle (*T*_m_) [the widening gray band shows the effect of using the minimum and maximum *T*_1-bar_ values, 1266 and 1447 °C, in Eq. (5)]. The fact that the gray band is quite narrow, even at 110 km (where *T*_m_–*T*_1-bar_ = ~192 °C) shows that a difference of 181 °C at 1 bar (1266–1447 °C), does not have a large effect on the calculated 1-bar olivine NiO content, even if the primary magma separates from its source at ~3.5 GPa (110 km). In contrast, variations in mantle olivine Ni content can have a large effect—the dashed lines reflect the uncertainty in the NiO content of the peridotite source (±0.06; 2 × MAD calculated using the midpoint temperature, 1356 °C). The error in $-\Delta _{r(1)} H_{{T_{\text{ref}} ,P_{\text{ref}} }}^{ \circ } /R$ can be ignored for present purposes (e.g., at a lithospheric thickness of 90 km, the propagated error on the calculated NiO from the uncertainty in $-\Delta _{r(1)} H_{{T_{\text{ref}} ,P_{\text{ref}} }}^{ \circ } /R$ is only ±0.005 wt%). The average calculated increase in the expected low-pressure olivine NiO content due to lithospheric thickness controlling the depth at which the primary magma separates from its mantle source coupled with the temperature dependence of $D_{\text{Ni}}^{\text{ol/liq}}$ is ~0.114 wt% per 100 km.

In Fig. 5, we compare our model 1-bar olivine NiO contents calculated as a function of the depth of primary melt segregation to observed nickel contents in olivine phenocrysts from MORBs and lavas from the Hawaiian–Emperor seamount chain (H-E), the Galápagos, the North Atlantic Igneous Province (NAIP; Iceland, West Greenland, and Baffin Island/Bay), Réunion, the Azores, and the Canary Islands (see caption for island abbreviations) plotted as a function of depth to the lithosphere–asthenosphere boundary (LAB) at the time of eruption (drawn largely from Dasgupta et al. 2010; other data sources are noted in Table S1). Olivine analyses for the Galápagos are from Vidito et al. (2013); for West Greenland and Baffin Island/Bay, data from Herzberg et al. (2016) were combined with those of Sobolev et al. (2007); analyses for the other localities are from Sobolev et al. (2007). Our model NiO olivine contents are calculated assuming no olivine crystallization as the primary magma migrates from its source region to the surface. It is, therefore, important to compare these calculated NiO values to those in the most primitive olivine phenocrysts at each of the localities as these primitive olivines are presumably closest in Ni composition to those that crystallized from the “primary” magmas. For each locality/suite of lavas, a plot of olivine Mg# versus olivine NiO defines either a relatively linear or curved trend. In order to estimate the “average” NiO content in the most primitive (i.e., highest Mg#) olivine in each of the suites of lavas, we fit each suite of data to either a linear or power law expression by minimizing the sum of the absolute deviations (in four of the 44 suites, samples defined two coherent trends and both were fit and in six of the 44, a subset of the samples defined a much more coherent trend in Mg#^ol^ versus NiO^ol^ space compared to the entire suite and, in these cases, only the subset was used; see notes to Table S1). The olivine with the highest Mg# in a given suite (or subset) was then used to calculate a NiO content (NiO_max-Mg#_) based on the regression equation for that suite—in this way, the plotted NiO content for each locality/suite of lavas (Fig. 5) reflects all or a large portion of the olivines analyzed for that suite (further details concerning the sample suites, e.g., the designations “tholeiitic,” “alkalic,” and “uncertain” in Fig. 5, are discussed in the notes to Table S1; regression coefficients are reported in Table S2).

Figure 5 shows that the NiO_max-Mg#_ values of olivine phenocrysts from MORBs and various OIBs are positively correlated with lithospheric thickness, consistent with the observation of Sobolev et al. (2007) and, although the scatter of the data points is large, suggesting that other factors are also at work, the positive correlation between lithospheric thickness and NiO_max-Mg#_ for the data plotted in Fig. 5 is significant at greater than the 99% confidence interval (Spearman rank-order correlation coefficient = 0.709). A weighted least-squares fit of olivine NiO_max-Mg#_ versus depth to the LAB using only those nickel contents from localities with maximum olivine Mg#s of ≥89 yields a slope per 100 km of 0.105 ± 0.006 wt% NiO. Note that the observed change in maximum NiO with increasing depth is consistent with the slope from the model *T*_1-bar_ = 1356 °C curve in Fig. 5 (0.114 wt% NiO/100 km) and, thus, the overall trend of increasing NiO in the most magnesian olivine phenocrysts in lavas erupted above lithosphere of increasing thickness is consistent with a temperature-dependent $D_{\text{Ni}}^{\text{ol/liq}}$ and a mantle source region (dominantly peridotite) with olivines that contain ~0.37 ± 0.06 wt% NiO. It is perhaps significant that both tholeiitic and alkalic basalts show a positive correlation in Fig. 5 suggesting that the same overall mechanism controls the Ni contents in both of these compositionally distinct groups of lavas. We want to emphasize that we are not asserting that source regions of the lavas shown in Fig. 5 are exclusively peridotitic in composition. The presence of pyroxene-rich lithologies has been widely invoked to account for aspects of the geochemical and isotopic variability in oceanic basalts (e.g., Hirschmann and Stolper 1996; Harpp and White 2001; Hofmann 2003; Huang and Frey 2005; Jackson et al. 2012; Lambart et al. 2013; Brown and Lesher 2014; Shorttle et al. 2014), and the presence of pyroxene-rich lithologies in the source regions may well contribute to some of the observed scatter in Fig. 5 (e.g., in samples from the Hawaiian–Emperor chain). Nevertheless, the positive correlation between NiO_max-Mg#_ and lithospheric thickness suggests an important role for a temperature-dependent $D_{\text{Ni}}^{\text{ol/liq}}$, as does the positive correlation between olivine NiO contents and olivine–spinel temperatures calculated for MORBs and basalts from large igneous provinces (Coogan et al. 2014). Finally, based on comparisons with olivines in Hawaiian lavas, Herzberg et al. (2016) suggested that the Ni contents of olivines in West Greenland and Baffin Island Paleocene picrites are not consistent with a temperature-dependent $D_{\text{Ni}}^{\text{ol/liq}}$. However, Fig. 5 shows that both the West Greenland and the Baffin Island/Bay data are, in fact, consistent with the model curve based on Eq. (5) (see Supplemental Information for a brief discussion of the apparent contrasting conclusions of Dasgupta et al. (2010) on the depth of melt equilibration for OIBs).

Even though the correlation between olivine NiO_max-Mg#_ and depth to the LAB is statistically significant, several of the MORB, Icelandic, and Galápagos suites plot below the dashed lower bound (−2 × MAD on the median mantle olivine NiO content). Furthermore, the *y*-intercept of the weighted fit to NiO_max-Mg# ≥ 89_ versus lithospheric thickness discussed above is 0.33 wt%, below the accepted median NiO content in peridotitic olivines (0.37 wt%), although still within two mean absolute deviations of the median value. Two possible explanations for these deviations are: (1) partial re-equilibration of the parental melts with the lithosphere as the liquids migrate to the surface and/or lithosphere erosion (both processes would move points to lower LAB depths in Fig. 5) and (2) not sampling the most primitive and thus highest Ni olivine in a suite of samples. Interactions between ascending parental melts and the lithosphere are likely. However, for Hawaii, both low- and high-silica magmas are observed (Rhodes and Vollinger 2004; Stolper et al. 2004) and compositions of melt inclusions in Hawaiian olivines are highly variable (e.g., Sobolev et al. 2000), suggesting that interactions of mantle melts with the lithosphere were insufficient to destroy their geochemical characteristics. In the remainder of this section, we focus on the second possible explanation—that of the vagaries of sampling.

Given that Ni is strongly partitioned into olivine and that olivine is generally the only silicate phase on the liquidus of primitive basalts at low pressures, the scatter to low Ni values in Fig. 5 may reflect the vagaries of sampling—i.e., the most Fo-rich olivines that crystallized from the parental magmas associated with a given suite were not analyzed (most of the low NiO data points in Fig. 5 are denoted by open symbols indicating that the maximum olivine Mg# for these suites is <90). There are at least two possible explanations for these anomalously low NiO_max-Mg#_ values that could reflect incomplete sampling:

- The first possibility is that the most primitive olivines are present in the lavas, although in low abundance, but are easily overlooked. This can be understood using the HSDP2 Mauna Kea data set of Sobolev et al. (2007), which contains 3869 olivine analyses from 42 polished thin sections. In 41 of these sections, there is no olivine analysis with an Mg# within 0.5 of the maximum value of 90.99 found in section SR277-8.0, and only 74 analyses (2%) have Mg#s within 1 unit of the maximum observed value (these 74 olivines are distributed among 11 thin sections). A simple Monte Carlo calculation indicates that had only three thin sections out of the 42 been analyzed (three being the median number of samples per suite/distinct population; see Table S1), then the likelihood of “capturing” the 90.99 value falls to ~7% and the probability of analyzing an olivine with an Mg# ≥90 is ~57% (assuming that all olivine phenocrysts are analyzed in each thin section). Note that the range of olivine compositions for many of the suites plotted in Fig. 5 is based on a small number of samples and a small number of analyses (i.e., not all available olivines in the chosen sections were analyzed). Thus, for those suites where the maximum observed Mg#^ol^ is less than ~90 and where a relatively small number of olivines were analyzed, it is plausible that the most primitive olivines present were missed.
- The second possibility is that the most primitive olivines were never erupted. Large piles of cumulate olivine are thought to lie beneath Hawaiian volcanoes (e.g., Clague and Denlinger 1994), and seismic refraction studies suggest that large volumes of gabbro exist beneath ocean islands that sit on thinner lithosphere (e.g., Galápagos; Richards et al. 2013). Furthermore, petrologic and geochemical arguments have been used to argue that high-pressure fractionation is an important process for MORBs from Knipovich Ridge (labeled “K” in Fig. 5, Hellevang and Pedersen 2005), in Iceland (e.g., Maclennan 2008; Winpenny and Maclennan 2011), and in the Galápagos (e.g., Geist et al. 1998). Therefore, magma chambers may act as density filters that prevent the earliest fractionation products (i.e., high-NiO olivines) from being erupted. Alternatively, based on extensive data from Icelandic lava flows, Thomson and Maclennan (2013) suggest that the majority of olivine phenocrysts are not in chemical equilibrium with their surrounding magma and that the olivine phenocrysts have resided in cumulate mush piles at the base of crustal magma chambers, where high NiO concentrations in olivine are likely to have been muted by diffusive re-equilibration [note that deformed olivine phenocrysts in picritic Hawaiian lavas have also been used to argue that they are not directly related to their entraining magma (e.g., Garcia 1996; Baker et al. 1996; Sakyi et al. 2012)]. Thus, magma chambers may act as density filters preventing primitive olivines from erupting and/or (given sufficient residence times) serving to homogenize olivine compositions through diffusive re-equilibration.

Since olivine residence times and entrainment processes are stochastic, most basaltic suites may contain olivines whose Ni contents either reflect or approach those values expected of olivines in equilibrium with the primary (or parental) magmas that have entered the system (e.g., Fig. 5)—although, as discussed above, the number of analyses required to discover olivines with these primitive Ni values may number in the hundreds to thousands. It is our hope that by using data sets with large numbers of analyses, and focusing on the NiO contents of the most Mg-rich olivine observed, we can minimize the effect of sampling “biases” of the sort described above.

**Fig. 6:**
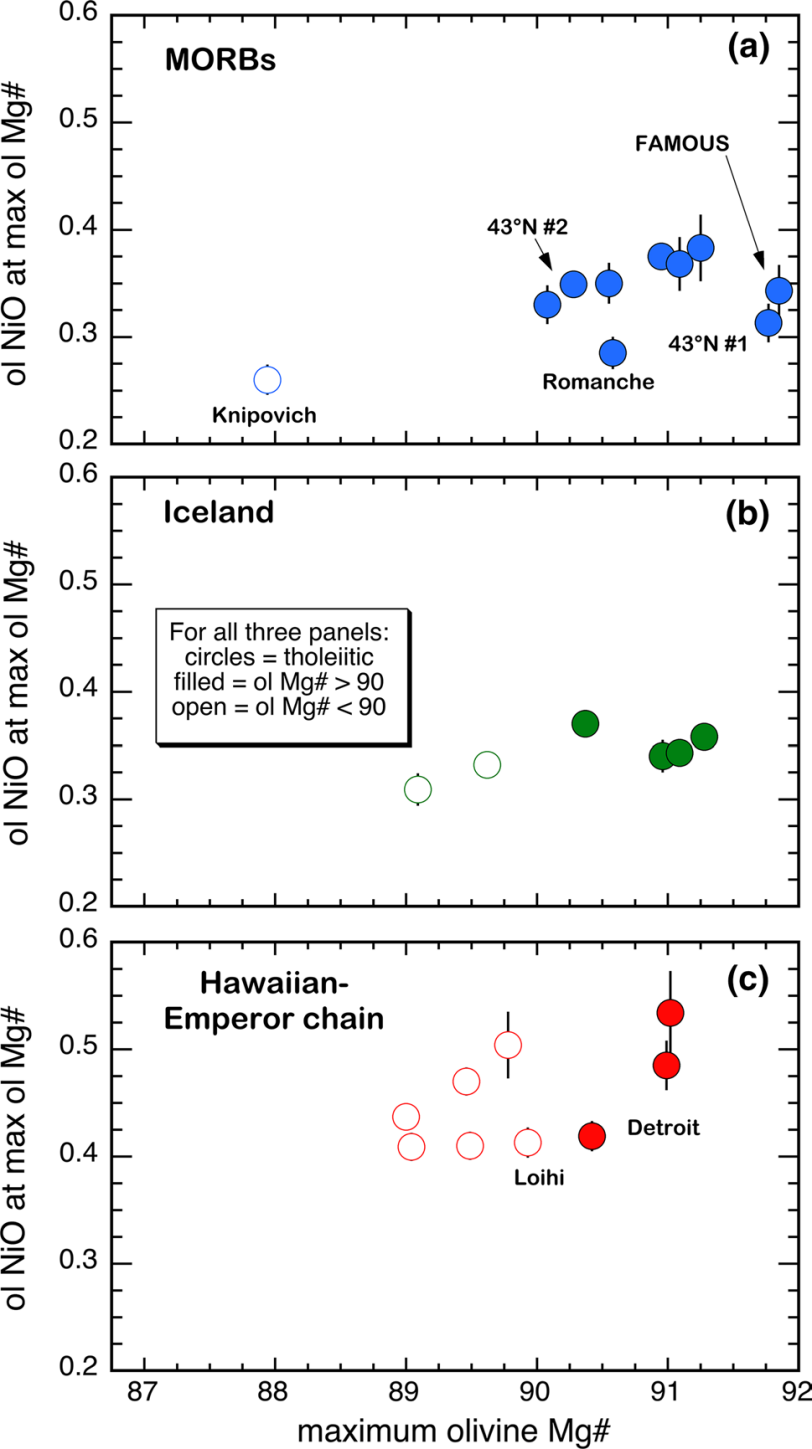
Calculated maximum olivine NiO contents (NiO_max-Mg#_; based on the regressions in Table S2) versus the maximum Mg#^ol^ observed at each locality. Only maximum NiO^ol^ values from suites of olivines from tholeiitic lavas are plotted. See text for further discussion

Finally, a potential test of the extent to which the various sampling and petrologic issues discussed above were operative is to plot NiO_max-Mg#_ values versus the maximum observed olivine Mg# for a given suite. Figure 6 shows that for MORBs and tholeiites from Iceland and Hawaii—those petrologic provinces where we have the most tholeiitic suites, the maximum olivine NiO and Mg# are positively correlated. If the most magnesian olivines in these low NiO^ol^ suites have not been sampled, the trends in Fig. 6 suggest that the low NiO_max-Mg#_ MORB, Icelandic, and Hawaiian suites might, thereby, project to higher NiO^ol^ values and become more consistent with the model curve of Fig. 5.

Given the factors discussed above that can skew compositions of entrained olivines in erupted lavas to lower Mg#s and nickel contents, the general agreement between the NiO contents of primitive olivine phenocrysts and the model curve shown in Fig. 5 is encouraging and consistent with lithospheric thickness controlling the depth at which melts last equilibrate with a largely peridotitic mantle.

## Conclusions

We performed experiments over a range of temperatures (1300–1600 °C) and pressures (1 atm–3.0 GPa) to separate the effects of temperature from those of liquid composition on the partitioning of Ni between olivine and silicate melt. The results of these experiments are subsets of glasses with approximately constant compositions (~12, ~15, and ~21 wt% MgO) coexisting with olivine. We parameterized our partitioning data using a Ni–Mg exchange reaction and, at 2 SE, the resulting temperature dependencies overlap with each other, with the temperature dependency of the ~18 wt% MgO^liq^ series determined by Matzen et al. (2013), and with a global set of data that spans a wide range of olivine and liquid compositions (this study, Matzen et al. 2013, and experiments from the literature). The relative insensitivity of $D_{\text{Ni}}^{\text{ol/liq}}$ obtained in this work to liquid composition makes the predictive model applicable to a wide range of natural melts.

The temperature dependence of $D_{\text{Ni}}^{\text{ol/liq}}$ causes the NiO contents of primitive near-surface olivine phenocrysts to scale with the temperature contrast between mantle melting and low-pressure crystallization, and if primary melts last equilibrate with the mantle near the lithosphere–asthenosphere boundary, then a correlation between NiO contents of initially crystallizing olivine and lithospheric thickness is a natural consequence of this temperature dependence of $D_{\text{Ni}}^{\text{ol/liq}}$. Our predictions compare favorably with the observed increase in NiO contents of high-Mg# olivine phenocrysts with lithospheric thickness, suggesting that these variations in observed NiO contents may reflect melting of and equilibration with mantle peridotite under lithospheric lids of varying thickness.

## Electronic supplementary material

Below is the link to the electronic supplementary material.

Supplementary material 1 (DOCX 3550 kb)

Supplementary material 2 (XLSX 19 kb)

Supplementary material 3 (XLSX 16 kb)
