# Supplementary material for: The effect of liquid composition on the partitioning of Ni between olivine and silicate melt
Source: Contrib Mineral Petrol. 2016 Dec 19;172(1):3. doi: 10.1007/s00410-016-1319-8 (PMC5165033; doi:10.1007/s00410-016-1319-8)
Supplement: Supplementary file 1 — Supplementary material 1 (DOCX 3550 kb) [file 410_2016_1319_MOESM1_ESM.docx]

Supplemental Material to the paper “The effect of liquid composition on the partitioning of Ni between olivine and silicate melt”

Andrew K. Matzen, Michael B. Baker, John R. Beckett, Bernard J. Wood, and Edward M. Stolper

**Evaluating deviations in**  **for each of the MgO series**

All of our experimental data from this study were used in global fits to equation (3) in the main text but we also examined series of experiments with roughly constant liquid compositions. However, as discussed in the main text, our choice of temperature and pressure for an experiment did not always produce a liquid composition with an MgO content that overlapped that of the target value (MgO*^liq,target^* = 12, 15, or 21 wt%, this study; or 18 wt% for Matzen et al. 2013). Since the effect of temperature on in each set of constant MgO experiments (the slope of the best-fit line in vs. *T*(°C) space for each of the series) can only be evaluated if variations in due to liquid composition (e.g., Hart and Davis 1978) are small, it is necessary to evaluate how deviations from MgO*^liq,target^* (and variations in olivine Mg#) among the experiments in a given series generate systematic offsets in the Ni partition coefficients. In practice, this means that runs with a relatively large deviation from the target MgO content should be excluded from the regression for that set of constant wt% MgO experiments.

To evaluate whether or not a compositional deviation was too large for an experiment to be assigned to one of the constant composition series, we compared, for each experiment, $D_{Ni,meas}^{ol/liq}$ (= NiO*^ol^*/NiO*^liq^*) to $D_{Ni,target}^{ol/liq}$ (= exp[3641/*T*(K) – 1.597 – ln(MgO*^liq,target^*/MgO*^ol^*)]) where 3641 and –1.597 are the –∆*H*/*R* and ∆*S*/*R* values from the weighted fit to all data from this study and Matzen et al. (2013), and NiO and MgO values for each phase are in wt%. Given observed variations in liquid MgO content and olivine Mg# within each set of constant MgO-series experiments, it’s easy to show that variations in MgO*^liq^* exert a much larger effect on than do observed variations in olivine composition. Thus, the difference between $D_{Ni,target}^{ol/liq}$and $D_{Ni,meas}^{ol/liq}$ shows how deviations in MgO content of the liquid from the target value in each experiment influence . Only for run 68 (this study) is $\left|D_{Ni,t\arg et}^{ol/liq}-D_{Ni,meas}^{ol/liq}\right|$ larger than twice the uncertainty calculated for the experimental $D_{Ni,meas}^{ol/liq}$ value using the mean fractional error calculated from all 28 $D_{Ni,meas}^{ol/liq}$ values (this study and Matzen et al. 2013) and their associated standard deviations. This approach of using a mean fractional error generates a standard deviation for each experimental $D_{Ni,meas}^{ol/liq}$ that reflects the precision of the entire data set. The large value of $\left|D_{Ni,t\arg et}^{ol/liq}-D_{Ni,meas}^{ol/liq}\right|$ for run 68, 1.13, compared to its calculated standard deviation of 0.39, is the reason that it was not included in the regression of the ~12 wt% MgO series experiments (Fig. 2, main text).

**Predictive models for**

An important characteristic for any predictive model is its ability to reproduce the data used in its construction. Recently, Herzberg et al. (2013, 2014) compared some of the available models of and concluded that a Jones-Beattie-type model (e.g., Jones 1984; Beattie et al. 1991) recovered the experimental data with the smallest error. To arrive at this conclusion, Herzberg et al. (2013) used a sum-of-squares statistic, $\sqrt{\frac{\sum\left(D_{Ni,calc}^{ol/liq}-D_{Ni,meas}^{ol/liq}\right)^{2}}{N-1}}$, where *N* is the number of experiments considered. This statistic is highly vulnerable to outliers and computed deviations are weighted on an absolute, not relative, basis, making it more sensitive to high values than low ones.

After the addition of the new work presented here, our un-filtered experimental database contains 463 experiments, a significant increase compared to the 271 experiments compiled by Herzberg et al. (2013). Only ~40% of the 463 experiments passed our filtering tests, suggesting that Herzberg et al. (2013) may have used a noisier data set. For these reasons, we calculated relative percent errors (defined as, $100\times\left|D_{Ni,calc}^{ol/liq}-D_{Ni,meas}^{ol/liq}\right|/D_{Ni,meas}^{ol/liq}$ where the vertical bars denote absolute value) for the Jones-Beattie model and equation (3) from the present study using the entire Filter B′ data set (as opposed to the edited data set discussed in the main text, so as to provide a more stringent test). For the Jones-Beattie model, we used two sets of parameters; one as given by Beattie et al. (1991), and a second obtained by refitting the Filter B′ data set using their model. Both equation (3) from this study and the Beattie et al. equation were fit using the same robust technique and  values were calculated using single-cation mole-fraction liquid and olivine compositions; average percent errors for the three fits are: 11.3, equation (3); 18.1, Beattie et al. (1991); 14.9, Beattie et al. (1991), refit.

Figure S1 shows the fraction of experiments from the Filter B′ data set whose error on the calculated partition coefficient is less than, or equal to, a given percent error. A superior predictive model will include a larger fraction of experiments at any given error, especially for those data points with the smallest errors (i.e., it will plot above other models in Fig. S1). In this study and in Matzen et al. (2013), careful microprobe work led to ~5% uncertainties on our measured s; we take this to be a rough estimate of the errors expected when analytical uncertainties are minimized and, thus, a model that reproduces all experimentally-measured partition coefficients within ~5% would be viewed as a complete success. As shown in Fig. S1, using equation (3), 37% of the 183 experiments in the Filter-B′ data set have calculated s within 5% of their measured values (for the 28 experiments from this study and Matzen et al. (2013) the value is 54%). Despite having the same number of fit parameters (two), the performance of the Jones-Beattie model is not as good; only ~20% of the experiments have calculated s within 5% of measured values using coefficients reported by Beattie et al. (1991). This rises to ~25% if their coefficients are refit using the Filter-B′ data set, still well below the 37% achieved using equation (3). The superior performance of the temperature-dependent exchange-reaction model is not limited to the lowest percent errors; at any given percent error below 42%, the exchange-reaction model successfully reproduces more of the data set (at least up to ~97% of the data) than does the Jones-Beattie-type approach.

Another way to judge model performance is by asking what percent error on is needed to account for a certain fraction of experiments in the database. For example, to account for 80% of the experiments in the database using the temperature-dependent exchange reaction requires an error on  of 16%, whereas the Jones-Beattie approach (using the coefficients from Beattie et al. 1991) requires a substantially larger error on , 28% (using the refit coefficients and the Jones-Beattie equation, the required error is ~22%). The Filter-B′ data set contains experiments with similar  values but different  values (due to the temperature dependence of ), and thus some of the error associated with the Jones-Beattie equation seen in Fig. S1 may reflect this mix of 1-atm and high-pressure experiments. Figure S2 shows % error vs. cumulative fraction for robust fits to equation (3), this study, and the Jones-Beattie equation using only the 136 1-atm experiments in the Filter-B′ data set. Again, equation (3), this study, fits a larger fraction of the data at relatively low errors than does the Jones-Beattie model.

Finally, although the correlation between  and is strong, both Jones (1984) and Beattie et al. (1991) acknowledge that a strict thermodynamic treatment of partitioning data using an exchange reaction leads to a linear relationship between  and  (with a y-intercept of zero), only if is constant. Our experiments suggest that this term is not constant because is nonzero. This conclusion is corroborated by comparing the partitioning of Ni to that of other divalent cations (e.g., Ca, Mn, Fe, Co); using the Jones-Beattie model, most other divalent cations have y-intercepts near zero (i.e., Table 1 from Beattie et al. 1991), whereas the y-intercept for Ni is not only non-zero (–3.66), it is nearly an order of magnitude larger in an absolute sense than the next largest value, −0.385 (Co). By allowing a non-zero intercept, the fit ceases to be firmly rooted in thermodynamics and becomes a linear fit to an observed correlation. Such correlations are useful and can provide useful insights but, without a mechanistic framework to help us interpret the observations, it can be difficult to correctly identify the variables that drive an observed variation. This issue can be greatly magnified when the underlying data set has many cross-correlated variables. As we, and many others have observed, many variables (notably, temperature, composition, and ) in the database of available Ni-partitioning experiments are strongly correlated with one another (e.g., Arndt 1977; Hart and Davis 1978; Takahashi 1978; Matzen et al. 2013). Thus, we urge caution in using a Jones-Beattie type expression for olivine-liquid Ni partitioning when modeling a process such as high-pressure mantle partial melting followed by low-pressure crystallization. During such a process, temperature and  may be decoupled.

**Conditions of mantle melting and heterogeneity: Conclusions from Ni contents of olivines vs. bulk rock geochemistry**

A significant finding of our work is that the temperature dependence of causes the formation of near-surface primitive olivine phenocrysts with NiO contents that should scale with thickness of the lithosphere through which they were erupted (assuming the temperature contrast between the mantle source and olivine saturation near the surface is controlled by lithospheric thickness). Our predictions compare favorably with the NiO contents of magnesian olivine phenocrysts, suggesting that variations in observed NiO contents may reflect melting of and equilibration with mantle peridotite under lithospheric lids of varying thickness. This finding appears to contrast with conclusions of Dasgupta et al. (2010) who, using the major-element thermobarometer of Lee et al. (2009), showed that, for a large fraction of island-averaged calculated primary melts, the mean pressure of melting was significantly deeper than the base of the lithosphere. There are several possible explanations for the apparent inconsistency but the most reasonable one is that, as discussed by Dasgupta et al. (2010), the calculated pressures of melting based on silica activity reflect mean melting pressures. The favored model of Dasgupta et al. (2010) posits a source region for OIBs that contains at least two components, one of which is peridotite—the additional component in low-silica alkaline OIB source regions is a carbonated eclogite; for “high-silica” OIB magmas like those erupted on Hawaii, the additional source component is volatile-free, silica-excess eclogite (i.e., subducted MORB). Since both of these basaltic components have solidus temperatures below that of fertile peridotite at high pressures (e.g., Pertermann and Hirschmann 2003; Lambart et al. 2009; Gerbode and Dasgupta 2010), the major-element composition of a primary magma represents the “average” of relatively deep partial melts from the basaltic component and shallower partial melts from the peridotite (arguably up to the lithosphere-asthenosphere boundary). However, a MORB-based basaltic component is expected to have a low bulk nickel content (Hofmann 1988; Gale et al. 2013), and thus, the NiO content of the parental magmas will be defined by lower pressure equilibration with and partial melting of the peridotite. This would lead to pressure estimates using the NiO contents of primitive olivines that are less than those based on silica activities.

**References**

Arndt NT (1977) Partitioning of nickel between olivine and ultrabasic and basic komatiite liquids. Year Book - Carnegie Institution of Washington (76):553–557

Beattie P, Ford C, Russell D (1991) Partition coefficients for olivine-melt and orthopyroxene-melt systems. Contrib Mineral Petrol 109:212–224

Dasgupta R, Jackson MG, Lee C-TA (2010) Major element chemistry of ocean island basalts — Conditions of mantle melting and heterogeneity of mantle source. Earth Planet Sci Lett 289:377–392

Gale A, Dalton CA, Langmuir CH, Su Y, Schilling J-G (2013) The mean composition of ocean ridge basalts. Geochem Geophys Geosys 14:489–518

Gee LL, Sack RO (1988) Experimental petrology of melilite nephelinites. J Petrol 29:1233–1255

Gerbode C, Dasgupta R (2010) Carbonate-fluxed melting of MORB-like pyroxenite at 2.9 GPa and genesis of HIMU ocean island basalts. J Petrol 51:2067–2088

Hart SR, Davis KE (1978) Nickel partitioning between olivine and silicate melt. Earth Planet Sci Lett 40:203–219

Herzberg C, Asimow PD, Ionov DA, Vidito C, Jackson MG, Geist D (2013) Nickel and helium evidence for melt above the core-mantle boundary. Nature 493:393–397

Herzberg C, Cabral RA, Jackson MG, Vidito C, Day JMD, Hauri EH (2014) Phantom Archean crust in Mangaia hotspot lavas and the meaning of heterogeneous mantle. Earth Planet Sci Lett 396:97–106

Hofmann AW (1988) Chemical differentiation of the Earth: the relationship between mantle, continental crust, and oceanic crust. Earth Planet Sci Lett 90:297–314

Hole MJ, Millett JM (2016) Controls of mantle potential temperature and lithospheric thickness on magmatism in the North Atlantic Igneous Province. J Petrol 57:417-436

Jones JH (1984) Temperature- and pressure-independent correlations of olivine/liquid partition coefficients and their application to trace element partitioning. Contrib Mineral Petrol 88:126–132

Lambart S, Laporte D, Schiano P (2009) An experimental study of pyroxenite partial melts at 1 and 1.5 GPa: Implications for the major-element composition of Mid-Ocean Ridge Basalts. Earth Planet Sci Lett 288:335–347

Larsen LM, Pedersen AK (2000) Processes in high-Mg, high-T magmas: Evidence from olivine, chromite and glass in Paleogene picrites from west Greenland. J Petrol 41:1071-1098

Lee C-TA, Luffi P, Plank T, Dalton H, Leeman WP (2009) Constraints on the depths and temperatures of basaltic magma generation on Earth and other terrestrial planets using new thermobarometers for mafic magmas. Earth Planet Sci Lett 279:20–33

Matzen AK, Baker MB, Beckett JR, Stolper EM (2011) Fe-Mg partitioning between olivine and high-magnesian melts and the nature of Hawaiian parental liquids. J Petrol 52:1243–1263

Matzen AK, Baker MB, Beckett JR, Stolper EM (2013) The temperature and pressure dependence of nickel partitioning between olivine and silicate melt. J Petrol 54:2521–2545

Pertermann M, Hirschmann MM (2003) Partial melting experiments on a MORB-like pyroxenite between 2 and 3 GPa: Constraints on the presence of pyroxenite in basalt source regions from solidus location and melting rate. J Geophys Res 108:2125

Takahashi E (1978) Partitioning of Ni^2+^, Co^2+^, Fe^2+^, Mn^2+^ and Mg^2+^ between olivine and silicate melts: compositional dependence of partition coefficient. Geochim Cosmochim Acta 42:1829–1844

**
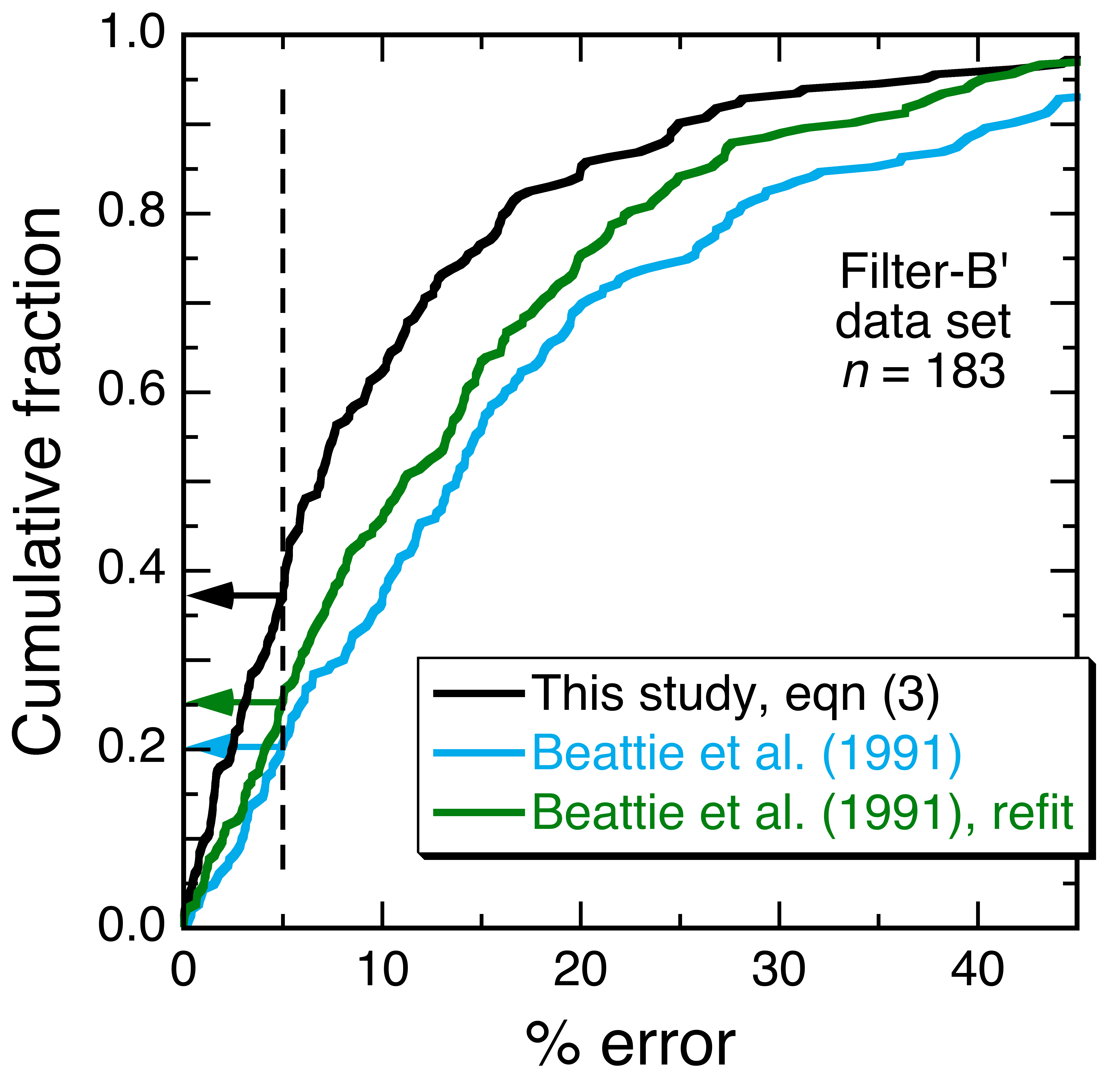
**

Fig. S1. Fraction of experiments whose relative percent errors ($100\times\left|D_{Ni,calc}^{ol/liq}-D_{Ni,meas}^{ol/liq}\right|/D_{Ni,meas}^{ol/liq}$, where vertical bars denote absolute value and “calc” and “meas” refer to calculated and measured, respectively), are less than or equal to a given percent error. For each experiment in the Filter-B′ data set, s were calculated using the exchange reaction (3) fit to the Filter- B′ data set (black curve); the Jones (1984) and Beattie et al. (1991) equation (= A× + B) and values for A and B from Beattie et al. (1991) (blue curve); and the same equation refit to the Filter-B′ data set (green curve). Horizontal arrows extend from each curve where it intersects a relative percent error of 5 (dashed line) and point to the fraction of the total number of experiments (*n* = 183) whose relative percent errors are ≤ 5. The thermodynamically-based exchange reaction (black curve) is better able to predict experimentally-measured s than a Jones-Beattie type model, despite both having the same number of free parameters.

**
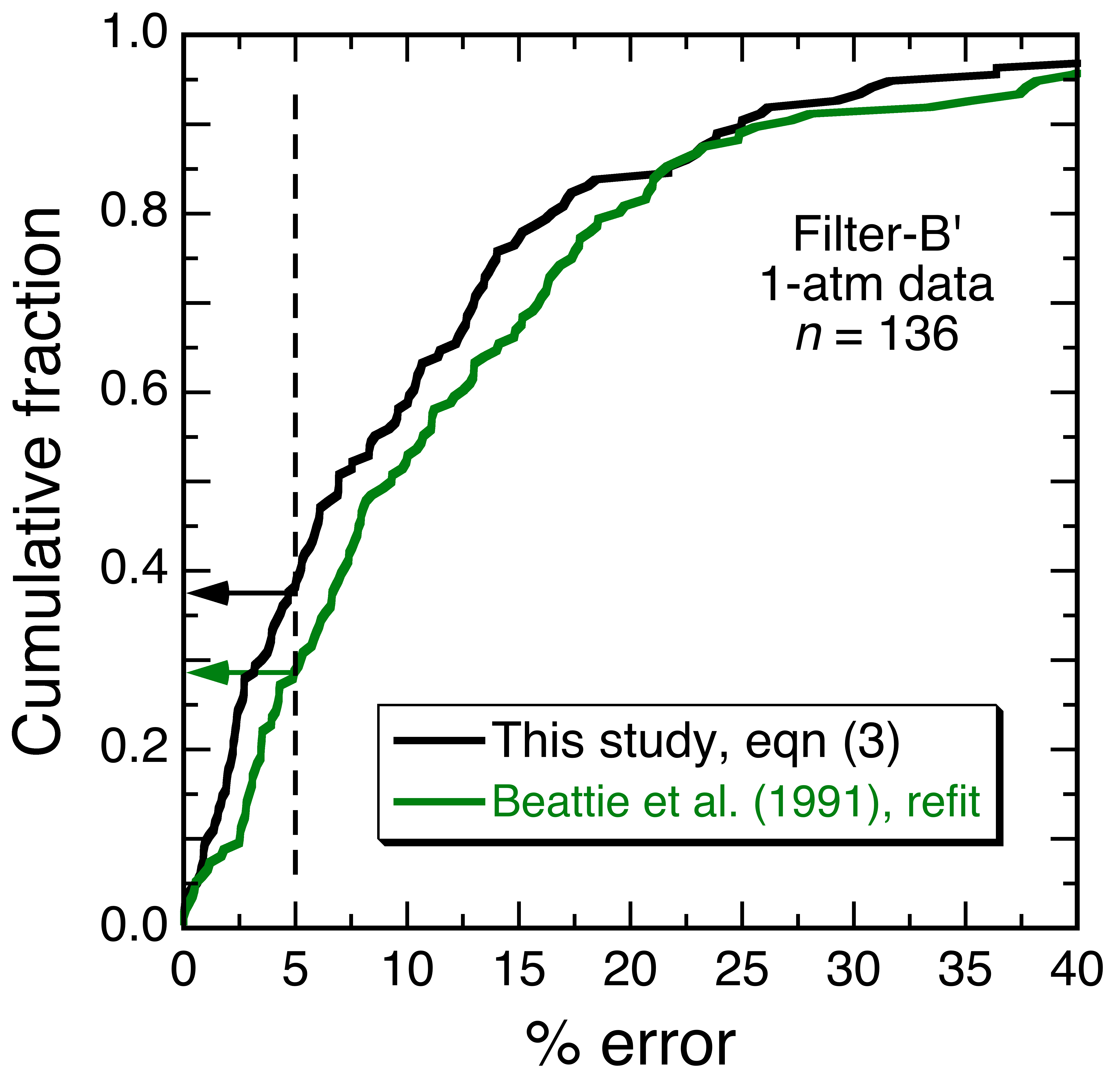
**

Fig. S2. Fraction of experiments whose relative percent errors, defined in the caption to Fig. S1, are less than or equal to a given percent error. For each 1-atm experiment in the Filter-B′ data set, s were calculated using the exchange reaction (3) fit to the 1-atm experiments in the Filter- B′ data set (black curve) and the Jones (1984) and Beattie et al. (1991) equation (= A× + B) refit to the 1-atm Filter-B′ experiments (green curve). Horizontal arrows extend from each curve where it intersects a relative percent error of 5 (dashed line) and point to the fraction of the total number of experiments (*n* = 136) whose relative percent errors are ≤ 5.

**Table S1**

-XLS file

**Table S2**

-XLS file
